# Trigonelline is an NAD^+^ precursor that improves muscle function during ageing and is reduced in human sarcopenia

**DOI:** 10.1038/s42255-024-00997-x

**Published:** 2024-03-19

**Authors:** Mathieu Membrez, Eugenia Migliavacca, Stefan Christen, Keisuke Yaku, Jennifer Trieu, Alaina K. Lee, Francesco Morandini, Maria Pilar Giner, Jade Stiner, Mikhail V. Makarov, Emma S. Garratt, Maria F. Vasiloglou, Lucie Chanvillard, Emilie Dalbram, Amy M. Ehrlich, José Luis Sanchez-Garcia, Carles Canto, Leonidas G. Karagounis, Jonas T. Treebak, Marie E. Migaud, Ramin Heshmat, Farideh Razi, Neerja Karnani, Afshin Ostovar, Farshad Farzadfar, Stacey K. H. Tay, Matthew J. Sanders, Karen A. Lillycrop, Keith M. Godfrey, Takashi Nakagawa, Sofia Moco, René Koopman, Gordon S. Lynch, Vincenzo Sorrentino, Jerome N. Feige

**Affiliations:** 1 Nestlé Institute of Health Sciences, Nestlé Research, Lausanne, Switzerland; 2 Nestlé Institute of Food Safety and Analytical Sciences, Nestlé Research, Lausanne, Switzerland; 3 Department of Molecular and Medical Pharmacology, Faculty of Medicine, University of Toyama, Toyama, Japan; 4 Centre for Muscle Research, Department of Anatomy and Physiology, University of Melbourne, Melbourne, Victoria Australia; 5 School of Life Sciences, Ecole Polytechnique Fédérale de Lausanne, Lausanne, Switzerland; 6 Mitchell Cancer Institute, Department of Pharmacology, F. P. Whiddon College of Medicine, University of South Alabama, Mobile, AL USA; 7 Institute of Developmental Sciences, Human Developmental and Health, Faculty of Medicine, University of Southampton, Southampton, UK; 8 National Institute for Health and Care Research, Southampton Biomedical Research Centre, University of Southampton and University Hospital Southampton NHS Foundation Trust, Southampton, UK; 9 Novo Nordisk Foundation Center for Basic Metabolic Research, Faculty of Health and Medical Sciences, University of Copenhagen, Copenhagen, Denmark; 10 Nestlé Health Science, Translation Research, Lausanne, Switzerland; 11 Mary MacKillop Institute for Health Research, Australian Catholic University, Melbourne, Victoria Australia; 12 Institute of Social and Preventive Medicine, University of Bern, Bern, Switzerland; 13 Chronic Diseases Research Center, Endocrinology and Metabolism Population Sciences Institute, Tehran University of Medical Sciences, Tehran, Iran; 14 Metabolomics and Genomics Research Center, Endocrinology and Metabolism Molecular-Cellular Science Institute, Tehran University of Medical Sciences, Tehran, Iran; 15 Singapore Institute for Clinical Sciences (A*STAR), Singapore, Singapore; 16 Department of Biochemistry, Yong Loo Lin School of Medicine, National University of Singapore, Singapore, Singapore; 17 Bioinformatics Institute, Agency for Science, Technology and Research (A*STAR), Singapore, Singapore; 18 Osteoporosis Research Center, Endocrinology and Metabolism Clinical Sciences Institute, Tehran University of Medical Sciences, Tehran, Iran; 19 Non-Communicable Diseases Research Center, Endocrinology and Metabolism Population Sciences Institute, Tehran University of Medical Sciences, Tehran, Iran; 20 KTP-National University Children’s Medical Institute, National University Hospital, Singapore, Singapore; 21 Biological Sciences, Faculty of Environmental and Life Sciences, University of Southampton, Southampton, UK; 22 Medical Research Council Lifecourse Epidemiology Centre, University of Southampton, Southampton, UK; 23 Division of Molecular and Computational Toxicology, Department of Chemistry and Pharmaceutical Sciences, Amsterdam Institute for Molecular and Life Sciences, Vrije Universiteit Amsterdam, Amsterdam, the Netherlands; 24 Healthy Longevity Translational Research Programme, Yong Loo Lin School of Medicine, National University of Singapore, Singapore, Singapore

**Keywords:** Ageing, Cell biology, Energy metabolism

## Abstract

Mitochondrial dysfunction and low nicotinamide adenine dinucleotide (NAD^+^) levels are hallmarks of skeletal muscle ageing and sarcopenia^1–3^, but it is unclear whether these defects result from local changes or can be mediated by systemic or dietary cues. Here we report a functional link between circulating levels of the natural alkaloid trigonelline, which is structurally related to nicotinic acid^4^, NAD^+^ levels and muscle health in multiple species. In humans, serum trigonelline levels are reduced with sarcopenia and correlate positively with muscle strength and mitochondrial oxidative phosphorylation in skeletal muscle. Using naturally occurring and isotopically labelled trigonelline, we demonstrate that trigonelline incorporates into the NAD^+^ pool and increases NAD^+^ levels in *Caenorhabditis elegans*, mice and primary myotubes from healthy individuals and individuals with sarcopenia. Mechanistically, trigonelline does not activate GPR109A but is metabolized via the nicotinate phosphoribosyltransferase/Preiss–Handler pathway^5,6^ across models. In *C. elegans*, trigonelline improves mitochondrial respiration and biogenesis, reduces age-related muscle wasting and increases lifespan and mobility through an NAD^+^-dependent mechanism requiring sirtuin. Dietary trigonelline supplementation in male mice enhances muscle strength and prevents fatigue during ageing. Collectively, we identify nutritional supplementation of trigonelline as an NAD^+^-boosting strategy with therapeutic potential for age-associated muscle decline.

## Abstract (web summary)

Trigonelline is identified as a human metabolite that can be used for NAD^+^ synthesis and is shown to improve muscle function in aged worms and mice.

## Main

Sarcopenia is the functional decline of skeletal muscle during ageing which impairs mobility and leads to loss of physical independence and disability^7^. Clinically, sarcopenia is characterized by the pathological decrease of muscle mass, strength and gait speed^3,8,9^, and arises from myofibre wasting and a combination of molecular and cellular hallmarks of ageing that collectively impair contraction^10–12^. Among these, mitochondrial dysfunction has a prominent role^1,3,13–17^, with decreased mitochondrial biogenesis, altered mitochondrial dynamics and proteostasis, and reduced mitochondrial respiration and ATP production being established drivers of muscle ageing phenotypes^3,18,19^. Given that intertissue cross-talk controls the availability of metabolic fuels for mitochondrial bioenergetics and influences muscle function and quality of life, research efforts have also characterized systemic contributions to sarcopenia. These include chronic low-grade inflammation via pro-inflammatory cytokines, altered metabolic fluxes via reduced circulating levels of anabolic amino acids and perturbations of glucose, vitamin, and lipid metabolism^20–25^.

The Multi-Ethnic Molecular determinants of Sarcopenia (MEMOSA) study recently identified mitochondrial dysfunction and declined NAD^+^ levels as prominent molecular hallmarks of sarcopenia in human cohorts from different geographies^3^. NAD^+^ is an essential coenzyme, derived from precursors of the vitamin B_3_ family, and a key cofactor for cellular and organismal metabolism. Mammals can produce NAD^+^ from different dietary precursors; these include nicotinamide riboside (NR) and nicotinamide mononucleotide (NMN), converted via the nicotinamide riboside kinase (NRK) pathway, nicotinic acid (NA), also known as niacin, which is metabolized via the nicotinate phosphoribosyltransferase (NAPRT)-dependent Preiss–Handler pathway, tryptophan used in the de novo pathway for NAD^+^ biosynthesis, and nicotinamide (NAM), which generates NAD^+^ via the nicotinamide phosphoribosyltransferase (NAMPT) salvage pathway^26^. NAD^+^ levels decline during ageing in several metabolic tissues in rodents and humans^1,17,27,28^, including in skeletal muscle where there is replicated clinical evidence of age-related NAD^+^ deficiency^2,3^. However, it is still largely uncharacterized whether alterations in muscle mitochondrial and NAD^+^ homeostasis are reflected in the circulating metabolome, and thus could be used to define clinical biomarkers and therapeutic interventions to manage later life muscle decline.

**Fig. 1:**
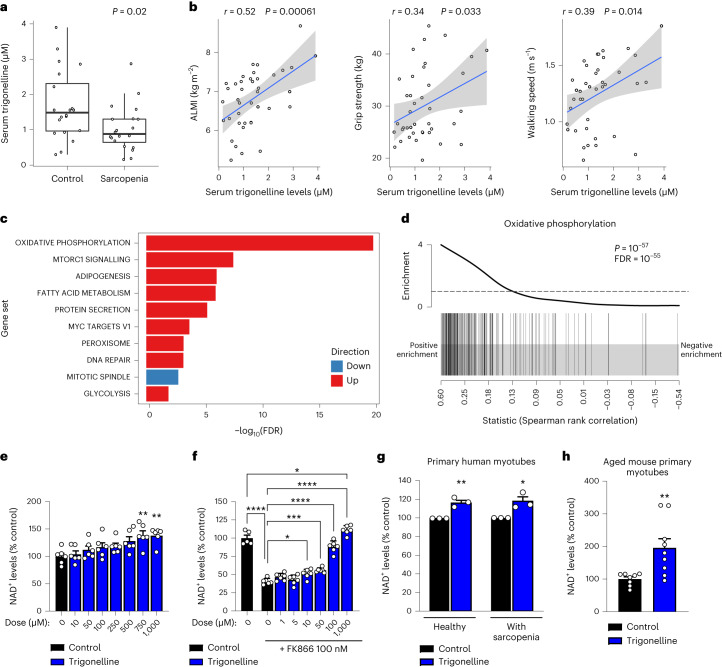
Serum trigonelline is reduced in human sarcopenia and is associated with mitochondrial and NAD^+^ metabolism in skeletal muscle. **a**, Serum levels of trigonelline in healthy controls (*n* = 20) and individuals with sarcopenia (*n* = 20) from the MEMOSA SSS (unpaired, two-tailed Student’s *t*-test). **b**, Association of serum trigonelline levels with ALMI, grip strength and gait speed; the Pearson correlation coefficient and its *P* value were calculated on *n* = 40 serum samples from the SSS. **c**, SSS muscle RNA-seq association with serum trigonelline levels. Gene set enrichment ordered according to the significance of enrichment with only the top ten gene sets being reported. A false discovery rate (FDR) < 10^−^^20^ was trimmed at FDR = 10^−^^20^ (*n* = 39 muscle samples). **d**, Enrichment plot for the hallmark oxidative phosphorylation gene set from **c**. **e**,**f**, Relative NAD^+^ levels in HSMMs after treatment with increasing concentrations of trigonelline, in the absence (**e**) or presence (**f**) of FK866 (one-way analysis of variance (ANOVA), mean ± s.e.m, *n* = 6 biological replicates per group). **g**, Relative NAD^+^ levels in human primary myotubes from healthy controls and patients with sarcopenia from the Hertfordshire Sarcopenia Study Extension (HSSe) cohort treated ex vivo with or without trigonelline (unpaired, two-tailed Student’s *t*-test, mean ± s.e.m, *n* = 3 biological replicates per group). **h**, Relative NAD^+^ levels in primary myotubes from aged mice (22 months) treated ex vivo with trigonelline (unpaired, two-tailed Student’s *t*-test, mean ± s.e.m, *n* = 8 and *n* = 9 biological replicates per group). *P < 0.05, **P < 0.01, ***P < 0.001, ****P < 0.0001. For the individual *P* values, see Fig. 1 in the Source data. Source data

**Extended Data Fig. 1:**
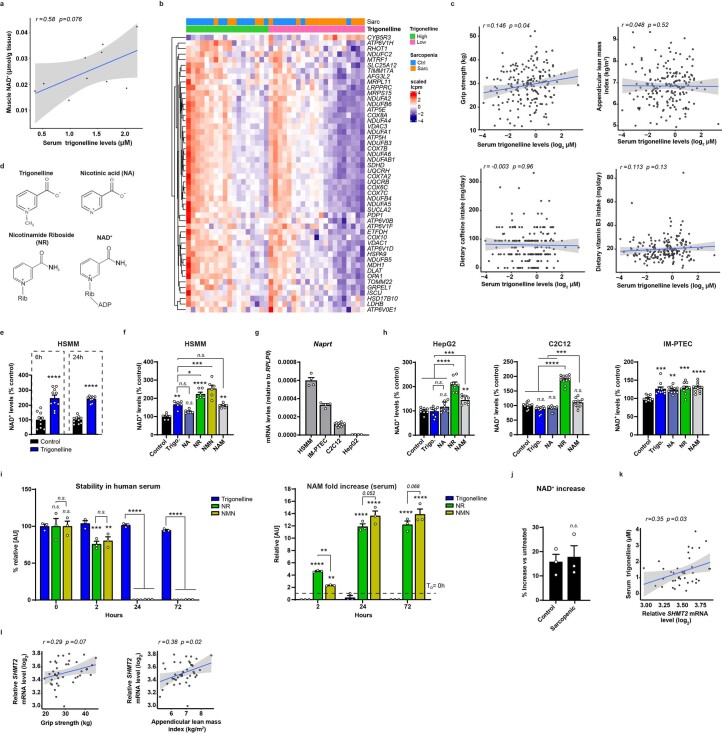
Trigonelline increases NAD^+^ in mammalian cells and its serum levels correlate with muscle NAD^+^ and Oxphos signatures in humans. **a**, Correlation of serum trigonelline levels with muscle NAD^+^, (n = 10 validation muscle samples remaining from the MEMOSA SSS cohort). **b**, Heatmap showing the top 50 oxidative phosphorylation genes associated with serum trigonelline levels. **c**, Correlation analysis of serum trigonelline levels with appendicular lean mass (ALM) index, grip strength, dietary caffeine and vitamin B3 intake in a replication cohort; Pearson correlation coefficient and its p-value were calculated on n = 186 serum samples from the Bushehr elderly health cohort. **d**, Molecular structures of nicotinic acid or niacin (NA), trigonelline which differs from NA only for the methyl group, and the ribosylated (rib) molecules nicotinamide riboside (NR) and NAD^+^. **e**, NAD^+^ levels relative to untreated cells measured in HSMM with or without trigonelline for 6 hours or 24 hours (unpaired two-tailed Student’s t-test, mean ± s.e.m, n = 10 biological replicates per group). **f**, NAD^+^ levels relative to untreated control measured in HSMM myotubes following 24 h NAD^+^ precursors treatment at 1 mM (One-way ANOVA; mean ± s.e.m, n = 6 biological replicates per group). **g**, *Naprt* mRNA expression in the indicated cell lines (n = 4–12 biological replicates per group). **h**, NAD^+^ levels relative to untreated control measured in HepG2, C2C12 and IM-PTECs cells following 24 h NAD^+^ precursors treatment (One-way ANOVA; mean ± s.e.m, n = 8–12 biological replicates per group). **i**, Stability assessment of trigonelline, NR and NMN added at 1 mM in human serum and incubated at 37 °C for the indicated durations (Two-way ANOVA; mean ± s.e.m, n = 3 biological replicates per group). **j**, Percentage increase in NAD^+^ levels in trigonelline treated primary myotubes from healthy control and sarcopenic HSSe subjects relative to untreated cells (unpaired two-tailed Student’s t-test, mean ± s.e.m, n = 3 biological replicates per group). **k**, Correlation analysis of serum trigonelline levels with *SHMT2* mRNA levels in muscle biopsies from the SSS cohort; Pearson correlation coefficient and its p-value were calculated on n = 39 serum samples. **l**, Correlation analysis of *SHMT2* gene expression in muscle biopsies with grip strength and appendicular lean mass index in the SSS cohort; Pearson correlation coefficient and its p-value were calculated on n = 40 serum samples. P: <0.05 (*); < 0.01 (**); < 0.001 (***); < 0.0001 (****); n.s., non-significant. For all the individual p values, see the Extended Data Fig. 1 Source file. Source data

To complement our previous analyses of muscle biopsies of human sarcopenia^3^ and understand if mitochondrial dysfunction and altered NAD^+^ metabolism could be linked to systemic changes, we investigated serum levels of the kynurenine / vitamin B metabolome in individuals with sarcopenia versus healthy controls from the MEMOSA cohort (Extended Data Table 1). No changes were observed during sarcopenia for most of the metabolites analysed, including the vitamin B_3_ forms that could act as potential NAD^+^ precursors. However, patients with sarcopenia had lower circulating concentrations of trigonelline, a natural alkaloid found in plants^4^ and animals, including in humans^29,30^ (Fig. 1a). Trigonelline levels were positively correlated with muscle mass assessed via Appendicular Lean Mass Index (ALMI) measured using dual-energy X-ray absorptiometry (DXA), grip strength and gait speed, all parameters used in the clinical definition of sarcopenia^3,9^ (Fig. 1b). Serum levels of trigonelline are also associated with NAD^+^ levels in skeletal muscle (Extended Data Fig. 1a), which we previously found to be linked with clinical measures of sarcopenia and muscle health^3^. Finally, gene set enrichment analysis identified a positive association between serum trigonelline levels and several metabolic and signalling pathways in skeletal muscle, with mitochondrial oxidative phosphorylation showing the strongest association with trigonelline (Fig. 1c,d and Extended Data Fig. 1b). Analysis of the Bushehr elderly health cohort^31^ (Extended Data Table 2) demonstrated that serum trigonelline is also associated with muscle function in an independent replication study (Extended Data Fig. 1c). Dietary records indicate that serum trigonelline levels are independent of dietary caffeine and vitamin B_3_ intake in this cohort, but possibly linked to other dietary factors, such as folate and fibre intake (Extended Data Fig. 1c and Extended Data Table 3). In addition, correction for dietary caffeine and vitamin B_3_ intake did not affect the association between trigonelline and muscle strength (Extended Data Table 4). Collectively, our targeted metabolomic profiling of human sarcopenia revealed trigonelline as a new metabolite associated with muscle function, mitochondrial metabolism and NAD^+^.

Trigonelline is an *N-*methylated form of NA (Extended Data Fig. 1d) that is synthesized by several plant species^4^ and is also a metabolite produced by the gut microbiome and endogenous metabolism in humans^29,30^. Based on this structural proximity to NA and the established link between muscle NAD^+^ and mitochondria in ageing and sarcopenia^1,14,15,17^, we tested if trigonelline could act as an NAD^+^ precursor, and directly impact NAD^+^, mitochondrial and muscle homeostasis. Primary human skeletal muscle myotubes (HSMMs) were treated with increasing therapeutic doses of trigonelline in the presence or absence of the NAMPT inhibitor FK866 (refs. ^14,32^) to block NAD^+^ salvage (Fig. 1e,f), thus mimicking low NAD^+^ via decreased *NAMPT* expression in human sarcopenic muscle^3^. Trigonelline increased NAD^+^ in control cells (half maximal effective concentration (EC_50_) = 315 µM) (Fig. 1e), fully rescued NAD^+^ deficiency in FK866-treated cells (EC_50_ = 110 µM) (Fig. 1f) and also increased NAD^+^ levels after prolonged treatment (Extended Data Fig. 1e). When compared to other NAD^+^ precursors in primary human muscle cells, trigonelline and the salvage precursor NAM induced NAD^+^ by approximately 50% while the NRK pathway precursors NR and NMN^33^ increased NAD^+^ levels approximately twofold (Extended Data Fig. 1f). The Preiss–Handler pathway of NAD^+^ biosynthesis requires the conversion of its substrate NA into NA mononucleotide (NAMN) via the rate-limiting enzyme NAPRT^6^. When we tested trigonelline and other precursors in additional cell lines of muscle, liver and kidney, trigonelline or NA failed to raise NAD^+^ levels in HepG2 (ref. ^34^) and C2C12 cells, which have low *NAPRT* expression (Extended Data Fig. 1g,h), while all precursors had similar efficacy in renal proximal tubular epithelial cells (PTECs) (Extended Data Fig. 1h). To further understand the biological activity of these precursors after in vivo absorption, we compared their stability in human serum. Trigonelline was remarkably stable in serum over 72 h, whereas NR and NMN rapidly disappeared within hours after conversion to NAM (Extended Data Fig. 1i). Given its lower serum levels in individuals with sarcopenia (Fig. 1a), we tested trigonelline in sarcopenic muscle cells. Treating primary myotubes from different donors with sarcopenia and aged-matched healthy controls^35^ raised cellular NAD^+^ (Fig. 1g) with comparable efficiency between groups (Extended Data Fig. 1j). Similarly, trigonelline also increased NAD^+^ levels in primary myotubes derived from aged mice (Fig. 1h).

Trigonelline is *N*-methylated on its pyridine ring and needs to be demethylated before entry into the Preiss–Handler pathway and pyridine *N*-ribosylated for NAD^+^ biosynthesis (Extended Data Fig. 1d). Because there is no trigonelline demethylase identified in plants or in mammals^36^, we explored potential candidates that could be linked to trigonelline demethylation by correlating serum trigonelline levels with the expression of genes from human sarcopenic muscle filtered using RNA sequencing (RNA-seq) for demethylating or methyltransferase activity (Extended Data Table 5). *SHMT2*, which is involved in mitochondrial one-carbon metabolism, had the strongest association with serum trigonelline (Extended Data Fig. 1k), and correlated positively with grip strength and muscle mass in humans (Extended Data Fig. 1l). While this observation uncovers a potential link between trigonelline and a methyltransferase involved in one-carbon metabolism that will require further exploration, SHMT2 is unlikely to directly demethylate trigonelline because it is not a *N*-methyltransferase and is known to cross-talk with NAD^+^ metabolism indirectly^37,38^.

**Fig. 2:**
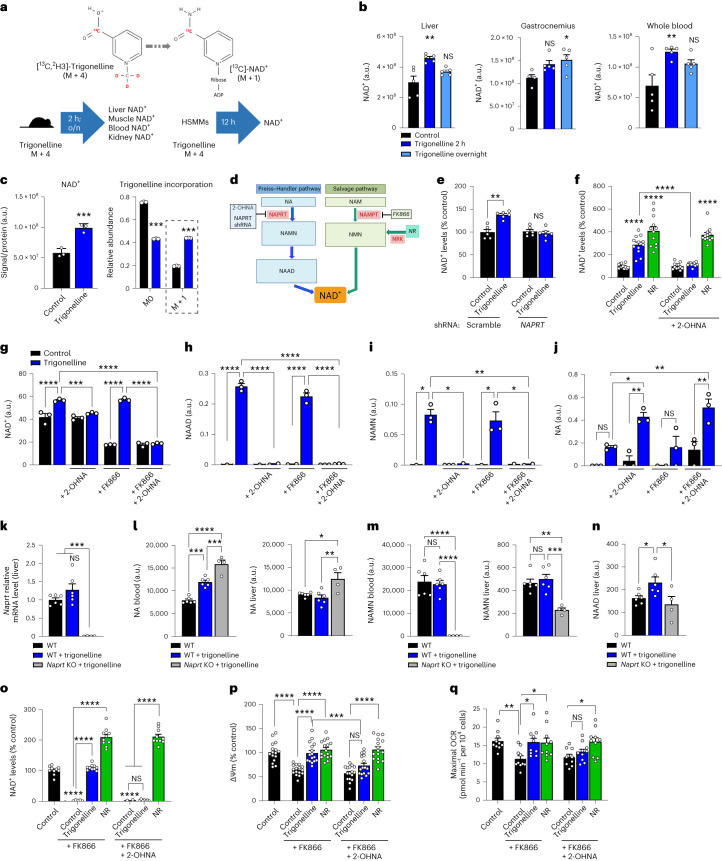
Trigonelline is an NAD^+^ precursor and activates mitochondrial function via the Preiss–Handler pathway. **a**, Experimental design of isotope-labelled trigonelline incorporation into NAD^+^. **b**, NAD^+^ levels measured using LC–HRMS in the liver, gastrocnemius muscle and whole blood 2 h or 16 h (overnight) after labelled trigonelline gavage in young mice (one-way ANOVA, *n* = 4–5 mice per group). **c**, NAD^+^ levels measured using LC–HRMS in HSMM (left) and relative isotopic enrichment of NAD^+^ (right) after 24-h incubation with 1 mM labelled trigonelline (unpaired, two-tailed Student’s *t*-test, *n* = 3 biological replicates per group). **d**, Representation of the Preiss–Handler and salvage pathways of NAD^+^ production. **e**, Relative NAD^+^ levels in HSMMs 48 h after adenoviral infection with a scrambled or *NAPRT* shRNA (unpaired, two-tailed Student’s *t*-test, *n* = 6 biological replicates per group). **f**, Relative NAD^+^ levels in HSMMs after 24-h trigonelline or NR treatment, with or without 2-OHNA co-treatment (one-way ANOVA, *n* = 12 biological replicates per group). **g**–**j**, NAD^+^ metabolites measured using LC–HRMS in HSMMs (NAD^+^, **g**; NAAD, **h**; NAMN, **i**; NA, **j**) after 24-h incubation with trigonelline in co-treatment with FK866, 2-OHNA or their combination (one-way ANOVA, *n* = 3 biological replicates per group). **k**, Quantitative PCR (qPCR) mRNA expression of *Naprt* in the liver of wild-type (WT) and *Naprt* knockout (KO) mice (one-way ANOVA, *n* = 4–6 animals per group). **l**–**n**, LC–HRMS measurement of NA (**l**) and NAMN (**m**) levels in the blood and liver, and of NAAD (**n**) in liver 2 h after trigonelline gavage in WT or *Naprt* KO mice (one-way ANOVA, *n* = 4–6 animals per group). **o**, Relative NAD^+^ levels in HSMMs after 72 h trigonelline or NR treatment, with or without co-treatment with FK866, 2-OHNA or their combination (one-way ANOVA, *n* = 10 biological replicates per group). **p**, Mitochondrial membrane potential (ΔΨm) measured using JC-1 staining in HSMMs treated as in **o** (one-way ANOVA, *n* = 16 biological replicates per group). **q**, Maximum oxygen consumption rate (OCR) in HSMMs treated as in **o** after stimulation with 3 μM carbonyl cyanide-*p*-trifluoromethoxyphenylhydrazone (one-way ANOVA, *n* = 9–10 biological replicates per group). All data are expressed as the mean ± s.e.m with *P < 0.05, **P < 0.01, ***P < 0.001, ****P < 0.0001. NS, not significant. Individual *P* values are reported in Fig. 2 of the Source data. a.u., arbitrary units. Source data

**Extended Data Fig. 2:**
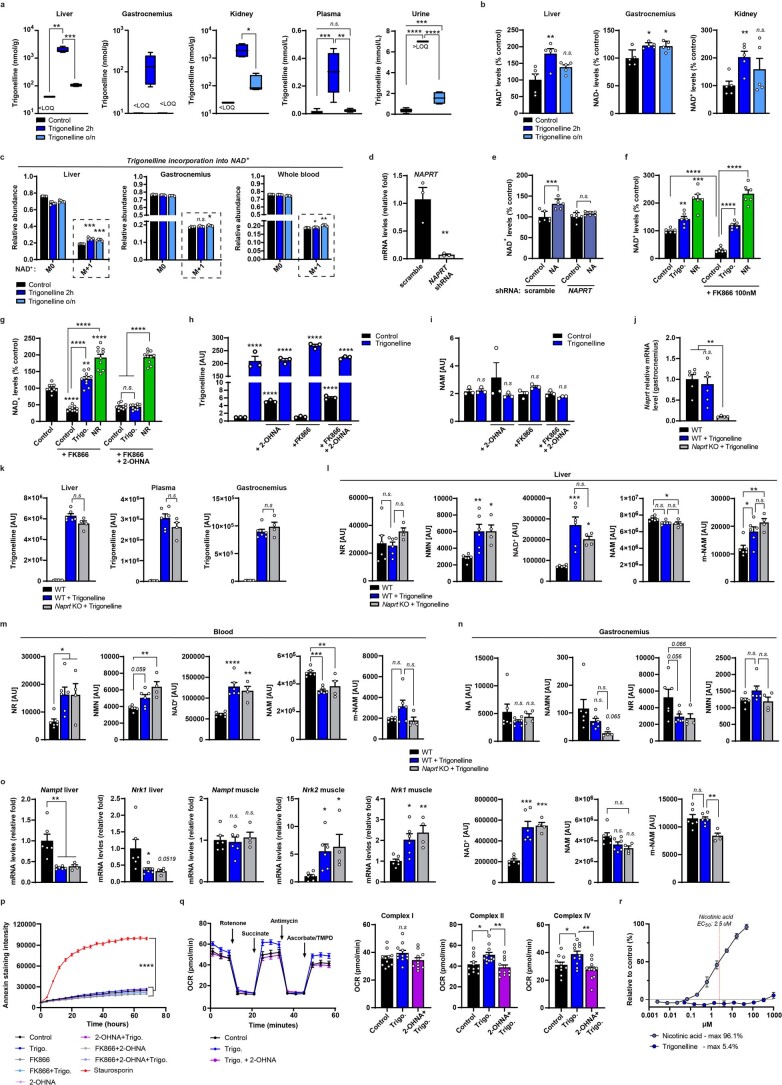
Trigonelline and Preiss-Handler pathway metabolomics and effects on NAD^+^ levels and mitochondrial homeostasis. **a**, LC-HRMS measurements of trigonelline levels in liver, gastrocnemius muscles, kidney, whole blood and urine of C57BL/6J mice, collected at 2 hours (2 h) or overnight (o/n) after labelled trigonelline gavage (unpaired two-tailed Student’s t-test compared to control group, mean ± s.e.m, n = 5 biological replicates per group, except in urine samples where only 2 samples were collected in controls and 4 in treated group); *LOQ*, level of quantification. **b**, NAD^+^ levels relative to untreated control mice measured in liver, gastrocnemius and kidney tissues from the same groups as in (**a**) (unpaired two-tailed Student’s t-test, mean ± s.e.m, n = 5 biological replicates per group). **c**, LC-HRMS-based relative trigonelline incorporation into NAD^+^ in the same tissues as in (**a**), related to the NAD^+^ measured in Fig. 2b (unpaired two-tailed Student’s t-test compared to control group, mean ± s.e.m, n = 5 biological replicates per group). **d**, *Naprt* mRNA levels by qPCR in HSMM cells following 48 h incubation with an adenovirus carrying either a scrambled or *NAPRT* shRNA construct (unpaired two-tailed Student’s t-test, mean ± s.e.m, n = 3 biological replicates per group). **e**, NAD^+^ levels relative to untreated control measured in HSMM myotubes following 24 h incubation with NA in presence or absence of the *NAPRT* shRNA construct (unpaired two-tailed Student’s t-test, mean ± s.e.m, n = 6 biological replicates per group). **f**, NAD^+^ levels relative to untreated control measured in HSMM myotubes following 24 h trigonelline or NR treatment, in the presence or absence of FK866 (one-way ANOVA; mean ± s.e.m, n = 6 biological replicates per group). **g**, NAD^+^ levels relative to untreated control measured in HSMM myotubes following 24 h trigonelline or NR treatment, in the presence of FK866 and with or without co-treatment with 2-OHNA (one-way ANOVA; mean ± s.e.m, n = 10 biological replicates per group). **h-i**, LC-HRMS based measurements of trigonelline and NAM in HSMM myotubes treasted as in Fig. 2g–j (One-way ANOVA, mean ± s.e.m, n = 3 biological replicates per group; in red ****, unpaired two-tailed Student’s t-test for comparisons in conditions without trigonelline); AU, abitrary units. **j**, *Naprt* mRNA expression by qPCR in gastrocnemius muscle of wildtype (WT) and *Naprt* knockout (KO) C57BL/6N mice (One-way ANOVA, mean ± s.e.m, n = 4–6 animals per group). **k**, LC-MS measurements of trigonelline levels in liver, plasma and gastrocnemius muscles harvested 2 hours post trigonelline gavage in WT or *Naprt* KO mice (unpaired two-tailed Student’s t-test, mean ± s.e.m, n = 4–6 animals per group); AU, abitrary units. **l-n**, LC-MS measurements of NAD^+^ metabolites in (**l**) liver, (**m**) blood, (**n**) gastrocnemius of the same groups as in (**k**) (One-way ANOVA, mean ± s.e.m, n = 4–6 animals per group); AU, abitrary units. **o**, *Nampt and Nrk* mRNA levels by qPCR in liver and muscle of the same groups as in (**j**) (One-way ANOVA, mean ± s.e.m, n = 4–6 animals per group). **p**, Apoptosis detection via annexing staining time course in HSMM myotubes following treatments of trigonelline, FK866 or 2-OHNA and their combinations. Staurosporin (2.5 µM) is used as a positive control for apoptosis induction (two-way ANOVA; mean ± s.e.m, n = 6 biological replicates per group). **q**, Seahorse-based substrate-driven OCR measurement in permeabilized HSMM myotubes following 24 h trigonelline treatment, in the presence or absence of 2-OHNA, and with a sequence of substrate/inhibitors to extract contribution of different complexes (one-way ANOVA; mean ± s.e.m, n = 11 biological replicates per group). **r**, GPR109A agonist assay in cells incubated with increasing doses of NA or trigonelline (mean ± S.D., n = 4). P: <0.05 (*); < 0.01 (**); < 0.001 (***); < 0.0001 (****); n.s., non-significant. For all the individual p values, see the Extended Data Fig. 2 Source file. Source data

To assess whether trigonelline is incorporated into the NAD^+^ molecule, we used an isotopically labelled form of trigonelline carrying a ^13^C on the carboxylic acid group and three deuterium (^2^H) atoms on the methyl group (-CD_3_) (Fig. 2a). Administration of isotopically labelled trigonelline in mice was highly bioavailable with high levels of the parent trigonelline molecule detected in the liver, gastrocnemius muscle, kidney, blood and urine 2 h after oral intake, and were largely cleared after an overnight wash-out (Extended Data Fig. 2a). This was mirrored by increased NAD^+^ content in the liver, muscle, kidney and whole blood, detected with either liquid chromatography coupled high-resolution mass spectrometry (LC–HRMS) (Fig. 2b) or an NAD enzymatic assay (Extended Data Fig. 2b). Based on our labelling strategy, direct incorporation of trigonelline into NAD^+^ implies the loss of the deuterated methyl group, leading to a ^13^C-labelled NAD^+^ structure with a mass of M + 1 (Fig. 2a). Treatment of HSMMs with labelled trigonelline significantly increased both total cellular NAD^+^ and [^13^C]-NAD^+^ M + 1 (Fig. 2c). In vivo, administration of labelled trigonelline also significantly enriched M + 1 NAD^+^ in the liver and whole blood and, to a smaller extent, in the muscle, with hepatic incorporation being faster probably because of a first-pass effect (Extended Data Fig. 2c). Altogether, these results demonstrate that trigonelline is a bona fide NAD^+^ precursor that is directly incorporated in NAD^+^ in cells and multiple tissues.

Given the similarity of trigonelline with NA and its ability to overcome NAD^+^ salvage inhibition (Fig. 1e,f), but also its inefficacy in low *NAPRT*-expressing cells (Extended Data Fig. 1g,h), we tested whether NAPRT would be required for the use of trigonelline, and used NR as a reference compound able to generate NAD^+^ independently of the Preiss–Handler pathway (Fig. 2d). Short hairpin RNA (shRNA)-mediated knockdown of *NAPRT* in HSMMs blocked the generation of NAD^+^ by trigonelline or NA (Fig. 2e and Extended Data Fig. 2d,e). Similarly, the NAPRT inhibitor 2-hydroxynicotinic acid (2-OHNA)^39^ blocked the conversion of trigonelline to NAD^+^ in HSMMs, but as expected did not impair the NAD^+^ boosting effect of NR (Fig. 2f). Conversely, various levels of cellular NAD^+^ depletion by blocking NAD^+^ salvage with FK866 for 24 h were rescued by trigonelline (Fig. 1f and Extended Data Fig. 2f), but not when co-treating with 2-OHNA (Extended Data Fig. 2g). LC–HRMS analysis also confirmed that 2-OHNA blocks increased NAD^+^ after trigonelline treatment (Fig. 2g), without affecting cellular trigonelline levels in the treated groups (Extended Data Fig. 2h). Of note, endogenous levels of trigonelline increased in cells treated with 2-OHNA in the absence of trigonelline treatment (Extended Data Fig. 2h), suggesting that there is an endogenous flux of trigonelline through NAPRT in muscle cells. After trigonelline treatment, the Preiss–Handler pathway-related metabolites nicotinic acid adenine dinucleotide (NAAD) and NAMN downstream of NAPRT^6,33^ were increased and blocked by NAPRT inhibition with 2-OHNA (Fig. 2h,i), whereas NA, which is upstream of NAPRT, was accumulated (Fig. 2j); the salvage pathway metabolite NAM did not change (Extended Data Fig. 2i). Acute dosing of trigonelline in wild-type (WT) and *Naprt* KO mice^40^ (Fig. 2k and Extended Data Fig. 2j) equally increased trigonelline in tissues (Extended Data Fig. 2k), distal NAD^+^ metabolite fluxes in the liver and blood (Extended Data Fig. 2l,m) and tissue NAD^+^ levels (Extended Data Fig. 2l–n). However, in *Naprt* KO mice treated with trigonelline, NA accumulated in the blood and liver (Fig. 2l), while NAMN was strongly downregulated (Fig. 2m); NAAD induction was blunted in the liver (Fig. 2n), similarly to what is observed in HSMMs (Fig. 2h,j). After trigonelline gavage, *Nampt* and *Nrk1* expression was downregulated in the liver, while *Nrk1* and *Nrk2* were upregulated in muscle in both treatment groups (Extended Data Fig. 2o). This suggests that trigonelline engages the Preiss–Handler pathway during first-pass metabolism and that secondary compensations through the NR–NMN–NRK pathway also contribute to NAD^+^ in *Naprt* KOs. Altogether, our in vitro and in vivo results indicate that trigonelline is metabolized through the Preiss–Handler pathway via NAPRT and promotes NAD^+^ biosynthesis via both direct and indirect mechanisms.

To compare the functional relevance of modulating NAD^+^ metabolism via trigonelline and NR through the NAPRT and salvage routes, we measured mitochondrial respiration and membrane potential in HSMMs where low NAD^+^ was modelled with FK866 for 72 h. Like the 24-h treatment (Extended Data Fig. 2g), trigonelline boosted NAD^+^ and this effect was blocked by NAPRT inhibition with 2-OHNA (Fig. 2o), while no detrimental effect on cell viability was observed (Extended Data Fig. 2p). Low NAD^+^ in response to FK866 lowered the mitochondrial membrane potential (Fig. 2p), which was fully restored by trigonelline and NR, while 2-OHNA abolished the effects of trigonelline but not NR (Fig. 2p). FK866 treatment also significantly decreased maximal mitochondrial respiration (Fig. 2q), which was also rescued by trigonelline but blocked when trigonelline was co-treated with 2-OHNA (Fig. 2q). In contrast, NR was still able to bypass NAPRT inhibition and increase respiration (Fig. 2q). Finally, respirometry performed with mitochondrial complex inhibitors revealed higher levels of complex II-driven and complex IV-driven respiration in trigonelline-treated cells, again dependent on NAPRT (Extended Data Fig. 2q). The clinical use of NA is limited by its effects on skin flushing caused by binding to the G protein-coupled receptor 109A (GPR109A)^41^. Therefore, we also tested whether trigonelline may activate this receptor using a GPR109A-overexpressing stable cell line coupled to β-arrestin-based detection, and validated the assay with NA (EC_50_ = 2.5 µM) (Extended Data Fig. 2r). Trigonelline did not activate GPR109A when tested up to 1 mM, a dose 400-fold higher than the EC_50_ of NA (Extended Data Fig. 2r), suggesting that it could be better tolerated than NA. Collectively, these results demonstrate that trigonelline functionally rescues NAD^+^ deficiency and confirm that trigonelline requires NAPRT and a functional Preiss–Handler pathway for physiological activity on mitochondrial bioenergetics.

**Fig. 3:**
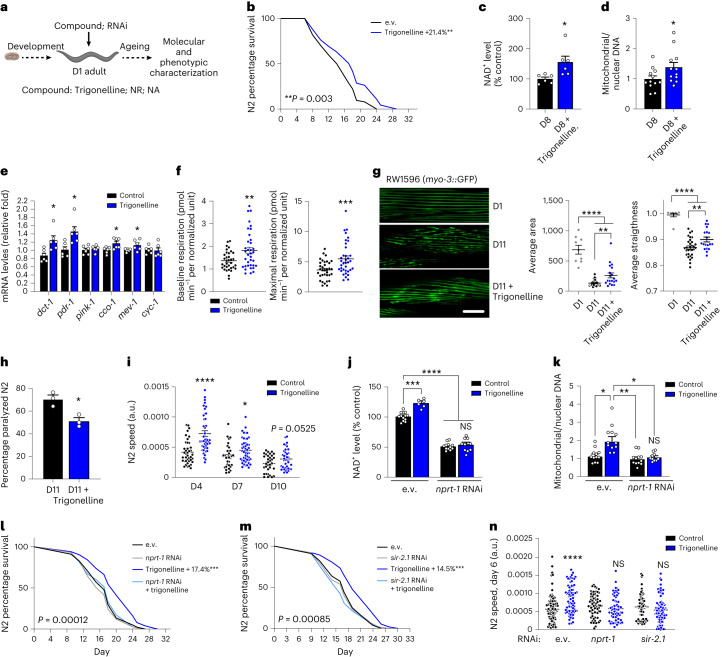
Trigonelline supplementation enhances the lifespan and ameliorates age-related muscle decline and mitochondrial dysfunction in *C. elegans*. **a**, Experimental design of compound treatments at 1 mM started from day 1 of adulthood (D1) in N2 WT worms. Illustration created with BioRender.com. **b**, Lifespan in trigonelline-treated worms (log-rank test, *n* = 90 worms per group). **c**, Relative NAD^+^ levels in aged worms on day 8 (D8). Unpaired, two-tailed Student’s *t*-test, *n* = 6 biological replicates per group). **d**, mitochondrial and nuclear DNA in aged worms (D8). Unpaired, two-tailed Student’s *t*-test, *n* = 12 worms per group). **e**, mRNA expression of mitochondrial genes in worms treated with trigonelline (unpaired, two-tailed Student’s *t*-test, *n* = 6 biological replicates per group). **f**, Basal and maximal OCR in L4 worms treated from the embryo stage (unpaired, two-tailed Student’s *t*-test, *n* = 36 animals per group). **g**, Confocal images (left) and quantitative integrity scoring (right) of green fluorescent protein (GFP)-labelled muscle fibres in adult (D1) and aged (D11) RAW1596 (myo-3p::GFP) worms (one-way ANOVA, *n* = 6 worms and 9–31 sarcomeres per group). Scale bar, 10 µm. **h**, Percentage of paralyzed aged worms (D11) (*n* = 3 independent experiments, unpaired two-tailed Student’s *t*-test). **i**, Spontaneous mobility of worms at different ages (unpaired, two-tailed Student’s *t*-test, *n* = 33–49 worms per group). **j**, Relative NAD^+^ levels in D1 adult worms treated from the embryo stage and fed with control (empty vector (e.v.)) or *nrpt-1* RNA interference (RNAi) (one-way ANOVA, *n* = 6–14 biological replicates per group). **k**, Mitochondrial and nuclear DNA in worms treated as in **j** (one-way ANOVA, *n* = 10–12 animals per group). **l**,**m**, Lifespan of control (e.v.), *nrpt-1* RNAi (**l**) and *sir-2.1* RNAi (**m**) worms (log-rank test, *n* = 100 animals per group). **n**, Spontaneous mobility of control (e.v.), *nrpt-1* RNAi and *sir-2.1* RNAi worms at D6 (unpaired, two-tailed Student’s *t*-test, *n* = 63–123 individual worms per group). All data are expressed as the mean ± s.e.m. with *P < 0.05, **P < 0.01, ***P < 0.001, ****P < 0.0001. Individual *P* values of the worms per group are reported in Fig. 3 of the Source data. Source data

**Extended Data Fig. 3:**
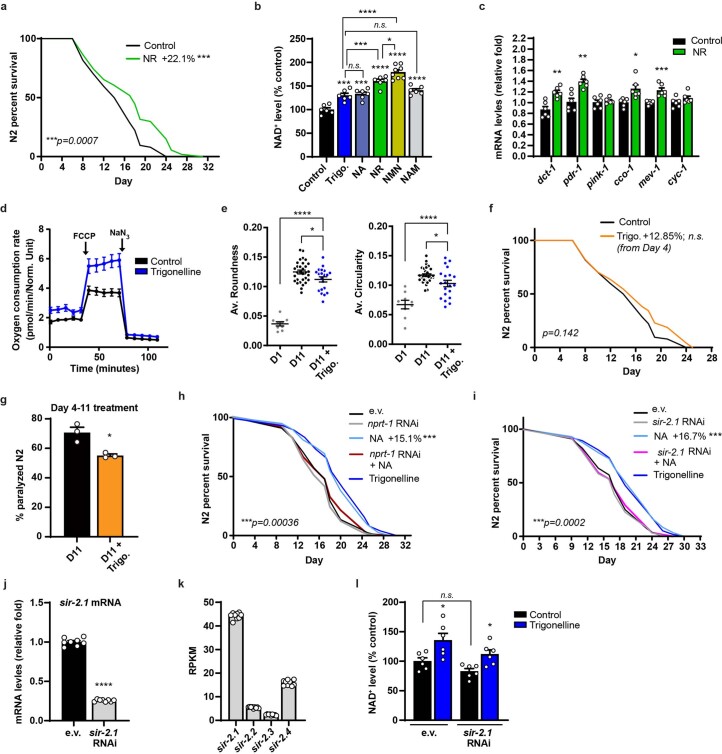
Effects of the NAD^+^ boosters trigonelline, NR and NA on nematode phenotypes. **a**, Lifespan assessment in N2 worms treated with or without NR as designed in Fig. 2a (log-rank test, n = 90 animals per group). **b**, NAD^+^ levels (% control) in D1 adult N2 worms treated with NAD^+^ precursors from embryo stage (One-way ANOVA, mean ± s.e.m, n = 6–8 biological replicates per group). **c**, mRNA levels by qPCR of genes regulating mitochondrial function in N2 worms treated with NR from Day 1 to Day 2 of adulthood (unpaired two-tailed Student’s t-test, mean ± s.e.m, n = 6 biological replicates per group). **d**, Seahorse-based oxygen consumption of L4 stage N2 worms from the Fig. 3f. **e**, Additional integrity scoring parameters of GFP-labeled muscle fibers in the worms from the Fig. 3g (One-way ANOVA, mean ± s.e.m, n = 6 animals, 9–31 sarcomeres per group). **f**, Lifespan of N2 worms treated with vehicle or trigonelline starting from Day 4 of adulthood (log-rank test, n = 90 animals per group). **g**, Percentage of paralyzed aged N2 worms (*D11=Day 11*) after vehicle or trigonelline treatment started at Day 4 of adulthood (n = 3 independent experiments, unpaired two-tailed Student’s t-test, mean ± s.e.m). **h**, Lifespan in N2 worms treated with or without NA or trigonelline and fed with control (*empty vector; e.v*.) or *nrpt-1* RNAi constructs from Day 1 of adulthood (comparing NA-treated worms to e.v., log-rank test, n = 100 animals per group). **i**, Lifespan in N2 worms treated with or without NA or trigonelline and fed with control (*e.v*.) or *sir-2.1* RNAi from Day 1 of adulthood (comparing NA-treated worms to e.v., log-rank test, n = 100 animals per group). **j**, *sir-2.1* mRNA levels by qPCR in D1 adult N2 worms fed with control (*e.v*.) or *sir-2.1* RNAi from embryo stage (unpaired two-tailed Student’s t-test, mean ± s.e.m, n = 8 biological replicates per group). **k**, sirtuin mRNA levels in N2 worms from publicly available RNA seq data^44^ (n = 9 replicates per group). **l**, NAD^+^ levels (% control) in D1 adult N2 worms treated with trigonelline from embryo stage and fed with control (*e.v*.) or *sir-2.1* RNAi constructs (One-way ANOVA, mean ± s.e.m, n = 6 biological replicates per group). P: <0.05 (*); < 0.01 (**); < 0.001 (***); < 0.0001 (****); n.s., non-significant. For all the individual p values, see the Extended Data Fig. 3 Source file. Source data

Lower NAD^+^ levels during ageing are linked to mitochondrial dysfunction, muscle decline and reduced fitness and lifespan in lower organisms, such as nematodes^14,42–45^. Given that NAD^+^-enhancing interventions can improve these phenotypes^14,42–45^, we next tested the impact of trigonelline during ageing in *Caenorhabditis elegans* by treating N2 WT worms starting from day 1 of adulthood (Fig. 3a). Trigonelline significantly extended the lifespan, with comparable effects to equimolar levels of NR (Fig. 3b and Extended Data Fig. 3a). Akin to that observed in human and mouse cells and tissues, trigonelline raised NAD^+^ levels in aged nematodes (Fig. 3c); its NAD^+^-boosting effects were comparable to NA and NAM, and partially lower than NR and NMN (Extended Data Fig. 3b). Trigonelline increased mitochondrial content assessed using the mitochondrial DNA/nuclear DNA ratio (Fig. 3d), activated the expression of genes linked to mitochondrial respiration and proteostasis (Fig. 3e), similar to NR (Extended Data Fig. 3c and^14,43^), and increased mitochondrial respiration (Fig. 3f and Extended Data Fig. 3d). We next evaluated muscle structure and mobility in ageing nematodes. Trigonelline supplementation improved myofibre integrity during ageing (Fig. 3g and Extended Data Fig. 3e); this was mirrored by reduced worm paralysis (Fig. 3h) and increased spontaneous mobility (Fig. 3i). Interestingly, worms treated with trigonelline later in adulthood showed only a mild lifespan extension (Extended Data Fig. 3f) but maintained better mobility than control animals during ageing (Extended Data Fig. 3g), indicating benefits of trigonelline on health span even when treating for a shorter duration and later in life. Importantly, the effects of trigonelline on NAD^+^ and mitochondrial content were lost with knockdown of the *Naprt* worm orthologue *nprt-1* (Fig. 3j,k), in line with what was observed in HSMMs. Knockdown of *nprt-1* or *sir-2.1* (Extended Data Fig. 3j), the predominant sirtuin in N2 worms (Extended Data Fig. 3k), both blunted the lifespan-extending effects and mobility benefits of trigonelline (Fig. 3l–n), in line with similar effects observed with NA (Extended Data Fig. 3h,i) and other NAD^+^ boosters^14,42–45^. As expected, trigonelline increased NAD^+^ levels after *sir-2.1* knockdown because sirtuins act as NAD^+^ sensors downstream of NAD^+^ biosynthesis and *sir-2.1* knockdown did not change baseline NAD^+^ levels (Extended Data Fig. 3l). Together, these results validate the physiological requirement of the Preiss–Handler pathway for longevity, mitochondrial and functional benefits of trigonelline in an in vivo model, and demonstrate that the observed benefits on ageing and health span require the NAD^+^-dependent sirtuin deacetylase.

**Fig. 4:**
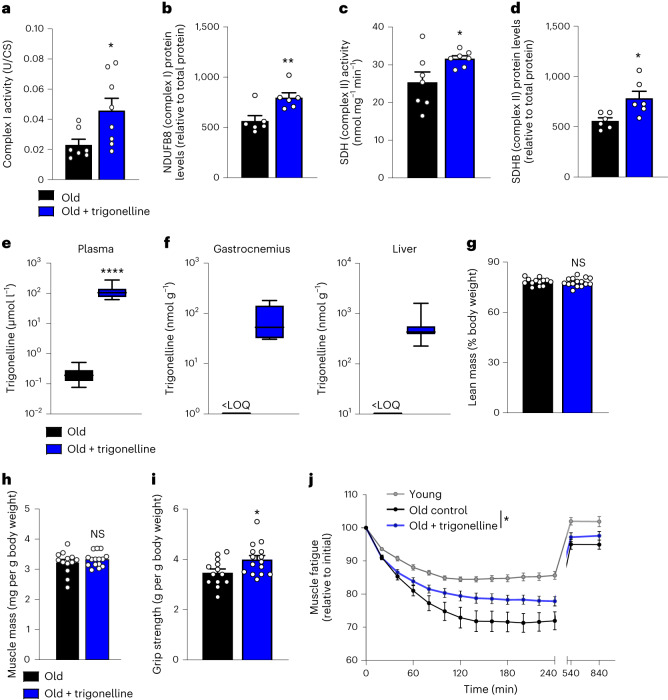
Trigonelline supplementation enhances mitochondrial activity and muscle function in aged mice. **a**, Mitochondrial complex I activity normalized to citrate synthase activity (UCI × UCS^−1^) in gastrocnemius muscle of aged mice (20 months) after a 5-day dietary supplementation of trigonelline (unpaired, two-tailed Student’s *t*-test, *n* = 7–8 biological replicates per group). **b**, Mitochondrial complex I (NDUFB8) protein levels in the same samples as in **a** (unpaired, two-tailed Student’s *t*-test, *n* = 6 biological replicates per group). **c**, Succinate dehydrogenase (SDH) activity in the quadriceps muscle of the same groups as in **a** (unpaired, two-tailed Student’s *t*-test, *n* = 7 biological replicates per group). **d**, Mitochondrial complex II (succinate dehydrogenase [ubiquinone] iron-sulphur subunit, mitochondrial (SDHE)) protein levels in the same samples as in **a** (unpaired, two-tailed Student’s *t*-test, *n* = 6 biological replicates per group). **e**, Plasma trigonelline levels measured in aged mice (22–24 months) after 12 weeks of trigonelline supplementation (unpaired, two-tailed Student’s *t*-test, *n* = 13 and 15 biological replicates per group). **f**, LC–HRMS measurement of trigonelline levels in gastrocnemius muscle and liver of the same mice groups as in **e** (unpaired, two-tailed Student’s *t*-test; gastrocnemius: *n* = 5 and 6, liver: *n* = 13 and 16 biological replicates per group); <LOQ, below the level of quantification. **g**, Lean mass normalized to body weight of aged mice after 12 weeks of treatment as in **e** (unpair**e**d, two-tailed Student’s *t*-test, *n* = 13 and 15 biological replicates per group). **h**, Tibialis anterior muscle mass of aged mice after 12 weeks of treatment as in **e** (unpair**e**d, two-tailed Student’s *t*-test, *n* = 13 and 15 biological replicates per group). **i**, Grip strength of aged mice after 12 weeks of treatment as in **e** (unpair**e**d, two-tailed Student’s *t*-test, *n* = 13 and 15 biological replicates per group). **j**, In situ muscle contractility normalized to initial force after supramaximal stimulation of the tibialis anterior muscle in young controls and aged mice after 12 weeks of treatment as in **e** (two-way ANOVA followed by uncorrected Fisher’s least significant difference tests; *n* = 11–14 biological replicates per group). All data are expressed as the mean ± s.e.m. with *P < 0.05, **P < 0.01, ***P < 0.001, ****P < 0.0001. Individual *P* values are reported in Fig. 4 of the Source data. Source data

**Extended Data Fig. 4:**
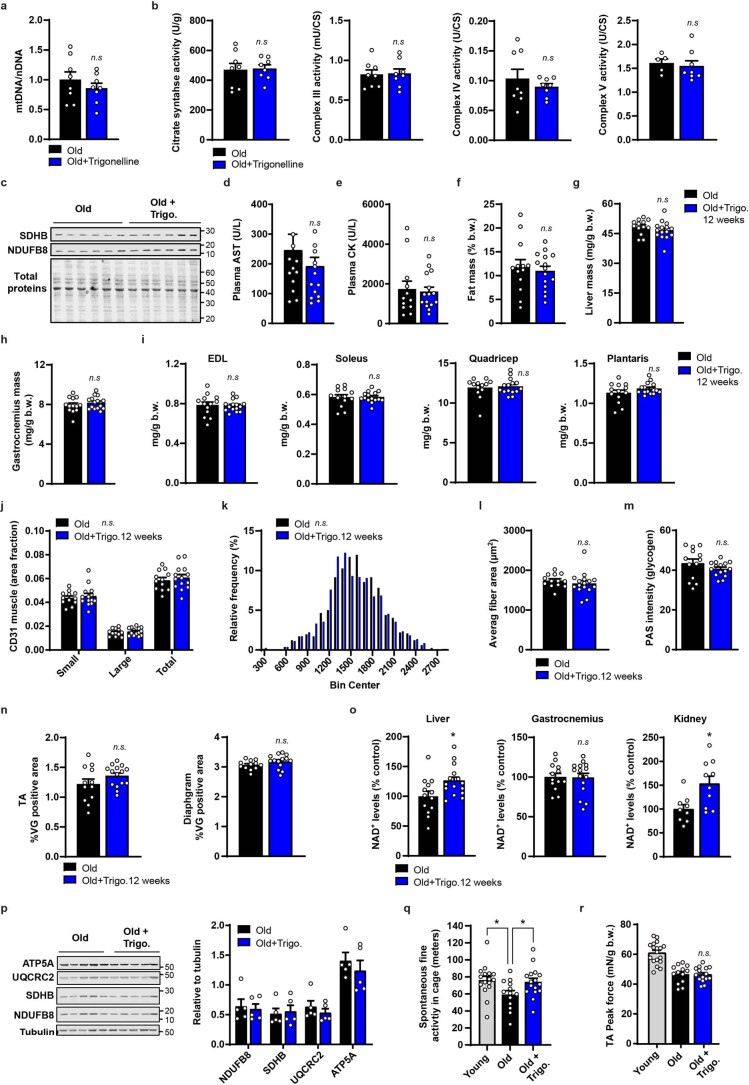
Effects of chronic trigonelline supplementation on physiological and muscle molecular readouts during aging. **a**, mt/nDNA of gastrocnemius muscle of aged C57BL/6J mice (20 months) following a 5 day diet supplementation of trigonelline (unpaired two-tailed Student’s t-test, mean ± s.e.m, n = 8 animals per group), related to Fig. 4a–d. **b**, Citrate synthase activity and additional enzymatic activity of Oxphos subunits from gastrocnemius muscles of the same groups as in (**a**) (unpaired two-tailed Student’s t-test, mean ± s.e.m, n = 8 animals per group, 3 samples in control group for complex V analyses could not be measured). **c**, Western blot of SDHB and NDFUB8 Oxphos subunits, and Licor staining for total proteins, quantified in Fig. 4b, d. **d**,**e** Plasma aspartate aminotransferase (AST) and creatine kinase levels measured in aged C57BL/6J mice (22–24 months) after a 12 week trigonelline supplementation (unpaired two-tailed Student’s t-test, mean ± s.e.m, n = 13 and 15 animals per group), related to Fig. 4e–j. **f**, Fat mass composition at the end of the intervention study normalized to body weight in the same groups as in (**d**) (unpaired two-tailed Student’s t-test, mean ± s.e.m, n = 13 and 15 animals per group). **g**, Liver mass at the end of the study normalized to body weight (unpaired two-tailed Student’s t-test, mean ± s.e.m, n = 13 and 15 animals per group). **h-i**, Muscle mass of additional muscles at the end of the study normalized to body weight (unpaired two-tailed Student’s t-test, mean ± s.e.m, n = 13 and 15 animals per group). **j**, Quantification of vascularization via CD31 immunostaining in TA muscle of the control and trigonelline-treated old mice (One-way ANOVA; mean ± s.e.m, n = 12 and 14 animals per group). **k**, Fiber size distribution in TA muscle of the same groups, with an average of 1543 ± 13 µm^2^ for trigonelline treated group and 1575 ± 13 µm^2^ for control group (Kolmogorov-Smirnov test, n = 13 and 16 animals per group). **l**, Average fiber area of TA muscle quantified in the same groups (unpaired two-tailed Student’s t-test, mean ± s.e.m, n = 13 and 16 animals per group). **m**, Quantification of PAS glycogen staining of TA muscles of the same groups (unpaired two-tailed Student’s t-test, mean ± s.e.m, n = 12 and 15 animals per group). **n**, Quantification of Van Gieson (VG) fibrosis staining of TA and diaphgram muscles of the same groups (unpaired two-tailed Student’s t-test, mean ± s.e.m, n = 12 and 15 animals per group). **o**, NAD^+^ levels relative to untreated old mice measured in liver, gastrocnemius and kidney tissues after chronic trigonelline administration (unpaired two-tailed Student’s t-test, mean ± s.e.m, n = 13 and 15 biological replicates per group for liver and muscle, n = 9 and 10 biological replicates per group for kidney). **p**, Western blot and related quantification of Oxphos protein levels in gastrocnemius muscle of the aged control and trigonelline-treated mice (unpaired two-tailed Student’s t-test, mean ± s.e.m, n = 5 biological replicates per group). **q**, Spontaneous fine activity of the aged control and trigonelline-treated mice, and of the young reference group (One-way ANOVA; mean ± s.e.m, n = 13 aged animals, and 16 animals for young and trigonelline groups). **r**, Peak force normalized to body weight of the same groups (unpaired two-tailed Student’s t-test, mean ± s.e.m, n = 13 and 15 biological replicates per group). P: <0.05 (*); < 0.01 (**); < 0.001 (***); < 0.0001 (****); n.s., non-significant. For all the individual p values, see the Extended Data Fig. 4 Source file. Source data

To translate the functional impact of trigonelline on muscle health from nematodes to mammals, we finally supplemented aged mice with dietary trigonelline. A 5-day treatment increased the expression and activity of mitochondrial oxidative phosphorylation complexes I and II in skeletal muscle (Fig. 4a–d and Extended Data Fig. 4c), without influencing mitochondrial abundance measured with mitochondrial/nuclear DNA and citrate synthase (Extended Data Fig. 4a,b). A 12-week dietary trigonelline supplementation increased plasma, liver and muscle levels of trigonelline in aged mice (Fig. 4e,f), with no signs of toxicity (Extended Data Fig. 4d,e). Body composition was not impacted by the treatment, with no changes in lean and fat mass distribution in trigonelline-treated mice (Fig. 4g and Extended Data Fig. 4e), no change of liver and muscle mass (Fig. 4h and Extended Data Fig. 4h,i) and no effects on tibialis anterior muscle histology assessed using myofibre size, capillary area, glycogen accumulation or fibrosis in tibialis anterior and diaphragm muscle (Extended Data Fig. 4j–n). We next measured NAD^+^ in different tissues, where chronic trigonelline exposure significantly increased NAD^+^ in the liver and kidney (Extended Data Fig. 4o). Elevation of muscle NAD^+^ (Fig. 2b and Extended Data Figs. 2b and 4o) and oxidative phosphorylation proteins (Fig. 4b,d and Extended Data Fig. 4p) were flattened in chronic versus acute exposure, probably because of long-term physiological compensation as observed in clinical studies in muscle with dietary administration of other NAD^+^ precursors^46,47^.

The effects of trigonelline on in vivo muscle function were assessed by recording spontaneous activity and measuring by grip strength and in situ tibialis anterior muscle contraction via electrical stimulation of the sciatic nerve to overcome any effects on volition. Chronic supplementation of trigonelline significantly increased the grip strength of the forelimb muscles in aged mice (Fig. 4i) and normalized age-related decline of fine spontaneous activity that mobilizes the hindlimb and forelimb muscles (Extended Data Fig. 4q). The maximum tetanic force of the tibialis anterior muscles was not impacted in aged trigonelline-supplemented mice (Extended Data Fig. 4r), probably because tetanic force relies on muscle mass and neuromuscular coupling, both of which were not affected by trigonelline. However, when the tibialis anterior muscles were repeatedly stimulated in situ to assess muscle fatigue from high-intensity contractions, the age-related decline of the force was attenuated in aged mice treated with trigonelline (Fig. 4j). In this setting, which mimics a physiological situation where the performance of healthy young mice decreases by 10% during the fatigue protocol, muscle fatiguability was tripled in aged mice but trigonelline prevented approximately 50% of this age-related decline. Together, these functional data indicate that chronic trigonelline administration mitigates muscle decline during ageing by stimulating mitochondria and increasing muscle performance and resistance to fatigue during high-intensity contraction.

Although there is a common trajectory of age-related muscle decline in all individuals, the rate and extent of loss of muscle mass and strength varies between older individuals; furthermore, the prominent factors that drive the inter-individual susceptibility to sarcopenia and disability versus healthy ageing are poorly understood for therapeutic intervention. Our previous profiling of muscle biopsies from patients with sarcopenia versus age-matched healthy controls revealed that mitochondrial dysfunction and reduced NAD^+^, two well-established molecular hallmarks of organismal ageing^11^, are prominent features of pathological progression and sarcopenia^3^. In this study, we expanded our initial analysis to a targeted serum metabolomic profiling of sarcopenia and discovered that the NA-related alkaloid trigonelline is a circulating metabolite correlating with muscle strength and gait speed in humans, and declines during sarcopenia. Trigonelline is a well-described plant metabolite. Its synthesis from NA via methylation confers protective and adaptive functions^4,48^, without an established reutilization as a Preiss–Handler substrate^49,50^. Consequently, trigonelline is particularly abundant in plant-derived food products, such as coffee beans and fenugreek seeds, which have been reported to modulate endogenous trigonelline levels in humans^4,51,52^. Although high coffee consumption has been associated with a lower prevalence of sarcopenia in some populations^53^, our correlation analyses did not detect an association between circulating trigonelline and dietary caffeine intake levels in a population from the Middle East, possibly because coffee consumption is low in this population. Serum trigonelline levels were also not correlated to dietary vitamin B_3_ intake, suggesting that methylation of dietary vitamin B_3_ does not directly control endogenous trigonelline. It is also unlikely that intake of coffee and niacin directly drives the link between trigonelline and sarcopenia as the association of serum trigonelline with muscle function was not markedly influenced by correction of dietary caffeine and vitamin B_3_ intake. Interestingly, food may still contribute to trigonelline metabolism as the dietary intake of folate and fibre was linked to circulating trigonelline levels. In addition, trigonelline can be produced by the microbiome. Trigonelline was proposed as a biomarker of metabolic health or physical fitness because urinary trigonelline levels decrease during obesity and increase in professional athletes^29,30^. Therefore, the association of trigonelline and sarcopenia probably has multifactorial contributions via a complex cross-talk between different food groups and endogenous metabolism.

Our work also describes trigonelline as an NAD^+^ precursor and demonstrates the benefits of its therapeutic dietary supplementation for mitochondrial function, muscle health and mobility during ageing. NAD^+^ boosting by trigonelline is conserved across different model organisms and primary human muscle cells from healthy individuals and donors with sarcopenia. Trigonelline treatment in aged mice increased different domains of muscle performance that mobilize both leg and arm muscles by improving both muscle fatigue and grip strength, which are important for healthy physical ageing. While the preclinical tests of muscle function used do not always directly translate to human performance, fatigue and grip strength are also well-established measures of sarcopenia and physical frailty^54,55^ and are commonly used to assess the pathological impairment of quality of life in older people in clinical practice. However, sarcopenia is a multifactorial disease where both muscle mass and function decline beyond pathological thresholds^7,8^. As trigonelline improved grip strength and fatigue independently of changes in muscle mass and maximal tetanic strength, we conclude that trigonelline cannot reverse all causes of sarcopenia and will have to be combined with other nutrients that support muscle mass, such as protein, vitamin D or omega 3 fatty acids, or for the nutritional management of sarcopenia^7^. At the cellular level, the benefits of trigonelline on muscle performance did not arise from structural adaptations in the skeletal muscle architecture, such as myofibre cross-sectional area, vascularization and fibrosis, but were linked to functional improvements of the mitochondrial respiratory activity of complexes I and II. Using inhibitors and genetic loss of function in vitro and in vivo, we showed that trigonelline is metabolized via the Preiss–Handler pathway and requires NAPRT to boost NAD^+^, stimulate mitochondrial metabolism and increase mobility and lifespan during ageing. Our work with tracers and metabolomics also indicates that trigonelline is demethylated before entering the flux of NAD^+^ biosynthesis, which is in line with early biochemical evidence that an enzymatic activity from the liver may demethylate trigonelline in mammals^36^. However, there is no known demethylase that catalyses trigonelline demethylation. Although the correlation we discovered between *SHMT2* expression and serum trigonelline, muscle mass and muscle strength suggests that trigonelline could cross-talk with the *S*-adenosyl-L-methionine-dependent methyltransferase activity of SHMT2 and folate/one-carbon metabolism, future studies are required to characterize the mechanisms of trigonelline demethylation and the identity of the trigonelline demethylase.

Our results add trigonelline to the list of established dietary NAD^+^ boosters. These molecules, including NR, NA, NAM and NMN, have shown preclinical potential for ameliorating ageing and chronic diseases in model organisms and have been investigated in recent human clinical trials focused on healthy ageing^46^, obesity^56^, mitochondrial disease^57^, neurodegeneration^58^ or insulin sensitivity^47^, with different outcomes. Our work highlights that the NAD^+^-boosting capabilities of different precursors vary across tissues and experimental models according to the relative activity of different branches of NAD^+^ biosynthesis. Trigonelline contributes to the NAD^+^ pool via a more indirect route than the ribosylated precursors NR and NMN, but our comparative studies demonstrate that trigonelline has similar cellular and physiological benefits to NA and NR in cells or nematodes, most probably because of its higher stability than ribosylated precursors. NA has lipid-lowering properties^33^ and provides benefits in mitochondrial myopathies with impaired NAD^+^ salvage^57^; however, it has limited tolerability as it induces skin flushing through dermal Langerhans cells because of GPR109A-dependent vasodilation^41,59^. Our results indicate that unlike NA, trigonelline does not activate GPR109A at physiological and therapeutic levels, and probably provides a more favourable therapeutic profile. While trigonelline primarily engages the Preiss–Handler pathway across models, in vivo supplementation also elevates NAD^+^ metabolites from other biosynthetic routes, supporting the inter-organ cross-talk between NAD^+^ pathways already observed in humans and rodents with NR supplementation^46,60^. In particular, trigonelline increases NR and NMN in the liver or serum both in WT and *Naprt* KO mice; these secondary NAD^+^ fluxes can contribute to the NAD^+^ pool in vivo in the absence of *Naprt*. This highlights the complexity of systemic NAD^+^ metabolism modulation and the need to study the interplay between NAD^+^ fluxes and to run future comparative studies with different precursors in specific models and in humans. The heterogeneity of NAD^+^ biosynthesis and metabolism also suggests that some physiological specificity may exist between different precursors across organs, supporting additional translational applications of trigonelline. Because of its high bioavailability and serum stability, trigonelline can reach the brain and impact cognitive performance in mice with Alzheimer’s disease^61^, therefore holding potential for neurocognitive benefits in addition to muscle health. In addition, trigonelline can protect from metabolic dysfunction and improve glucose tolerance both in mice and humans^62,63^.

In summary, clinical profiling revealed an association between trigonelline and muscle health in humans. Our preclinical experiments demonstrated that trigonelline is an NAD^+^ precursor that optimizes mitochondrial function to improve muscle strength and prevent fatigue during ageing. Therefore, trigonelline is a nutritional geroprotector with therapeutic potential to manage sarcopenia and other age-related pathologies.

## Methods

### Human studies

Detailed description of the MEMOSA study is available in the study by Migliavacca et al.^3^. Twenty Chinese male participants aged 65–79 years with a diagnosis of sarcopenia and 20 healthy age-matched controls were recruited in Singapore under the National Healthcare Group Domain-Specific Research Board study (no. 2014/01304); each participant gave written informed consent. Muscle biopsy samples were fully depleted during the study analyses.

The cohort of the Bushehr nutritional epidemiology study in older people consists of 186 older men aged 60 years and older participating in the second stage of the Bushehr elderly health programme. They were randomly selected from the population-based prospective cohort study conducted in Bushehr, a southern province of Iran^31^. Grip strength was measured using a digital dynamometer with three repeats of each hand, and retaining the highest average handgrip strength for the strongest hand. The ALMI was measured using DXA (Discovery Wi), with the AMLI calculated for each participant as the sum of the upper and lower limb lean mass, expressed in kilograms, divided by the height square, expressed in metres. Dietary intake assessment was performed using a 24-h dietary recall of all food and beverages consumed, performed by expert nutritionists^31^. Standard reference tables were used to convert household portions to grams and quantified using the nutritionist IV package, modified for Iranian foods to obtain daily energy, nutrient values and servings of foods consumed. For mixed dishes, food groups and nutrients were calculated according to their ingredients. The study was approved by the Research Ethics Committee of the Endocrinology & Metabolism Research, Tehran University of Medical Sciences, under reference TUMS.EMRI.REC.1394.0036; and each participant gave written informed consent. Local sample analyses were approved by the cantonal ethics commission for human research (Commission Cantonale d’Éthique de la Recherche sur l’Étre Humain) in Vaud, Switzerland under reference 490/14. For both studies, tryptophan and vitaminB_3_ metabolites were measured in overnight fasting venous blood samples using liquid chromatography–tandem mass spectrometry as reported in Panahi et al.^64^ and Midttun et al.^65^.

Primary myotubes were derived from muscle biopsies from participants in the HSSe with approval from the Hertfordshire Research Ethics Committee^66^. Each participant gave written informed consent.

### Human RNA-seq and pathway analysis

RNA-seq of vastus lateralis muscle biopsies of the MEMOSA Singapore Sarcopenia Study (SSS) study was performed on libraries generated from 250 ng of total RNA using paired-end sequencing on an Illumina HiSeq 2500 sequencer at a depth more than 75 million reads per sample, as described previously^3^. All statistical analyses of the transcriptomics data were performed using R v.3.3.3 and relevant Bioconductor packages (for example, edgeR v.3.16.5). Briefly, after excluding one sample with an abnormally low percentage of uniquely mapped reads and after removing genes with a mean expression lower than 20 reads, data were normalized by the trimmed mean of M-values as implemented in the edgeR^67^ function calcNormFactors. Normalized skeletal muscle mRNA expression was associated with the serum level of trigonelline using Spearman rank correlation. The full dataset of genes expressed in skeletal muscle was used for pathway enrichment. Pathway enrichment analysis was performed using the Molecular Signature Database v.5.2 collections H (hallmark gene sets) and the mean-rank gene set enrichment test^68^ to assess whether an annotated set of genes was enriched in genes associated with serum levels of trigonelline. For Extended Data Table 5, the list of genes with mRNA expression correlating with serum trigonelline was filtered for genes annotated with demethylase or methyltransferase activity.

### Cell cultures and reagents

Human primary myoblasts from male donors (catalogue no. CC-2580, Lonza) were seeded in 96-well plates at a density of 12,000 cells per well in skeletal muscle growth medium (SKM-M0, AMSBIO). After 1 day, differentiation was performed by changing to DMEM/F12 (Thermo Fisher Scientific) supplemented with 2% horse serum for 4 days. Primary human myoblasts from male donors from the HSSe cohort (*n* = 3 with sarcopenia and *n* = 3 controls) were seeded in 96-well plates coated with Matrigel at a density of 15,000 cells per well in myoblast proliferation medium (DMEM, 20% FCS, 10% horse serum, 1% penicillin-streptomycin, 1% chick embryo extract). After 48 h, differentiation was induced by changing to differentiation medium (DMEM, 2% horse serum, 1% penicillin-streptavidin). HepG2 cells (catalogue no. HB-8065, ATCC), were maintained in exponential growth phase in DMEM supplemented with 10% FCS, at 37 °C in a humidified atmosphere of 5% CO_2_. C2C12 myoblasts (catalogue no. CRL-1772, ATCC) were cultured in a 5% CO_2_ incubator in high-glucose DMEM (Gibco) containing 10% FCS, and were differentiated into myotubes in high-glucose DMEM containing 2% horse serum when cell density reached 80–90% at 37 °C. Primary myotubes from aged male C57BL/6JRj mice (24 months; Janvier Labs) were generated from freshly sorted muscle stem cells isolated using flow cytometry as described previously^69^. Briefly, hindlimb muscles were rapidly collected, minced and digested with 2.5 U ml^−1^ Dispase II (Sigma-Aldrich), 0.2% Collagenase B (Sigma-Aldrich) and 5 mM MgCl_2_ at 37 °C. The preparation was then filtered sequentially through 100-μm and 30-μm filters and cells were incubated at 4 °C for 30 min with antibodies against CD45 (1:25 dilution, catalogue no. MCD4528, Invitrogen), CD31 (1:25 dilution, catalogue no. RM5228, Invitrogen), CD11b (1:25 dilution, catalogue no. RM2828, Invitrogen), CD34 (1:60 dilution, catalogue no. 560238, BD Biosciences), Ly-6A/E (1:150 dilution, catalogue no. 561021, BD Biosciences) and α7-integrin (1:30 dilution, catalogue no. FAB3518N, R&D Systems). Muscle stem cells identified as CD31^−^CD11b^−^CD45^−^Sca1^−^CD34^+^integrin α7^+^ were isolated with a Beckman Coulter Astrios Cell sorter. Fluorescence-activated cell sorting-isolated muscle stem cells were plated onto gelatin-coated 384-well plates at a density of 600 cells per well in DMEM supplemented with 20% heat-inactivated FCS (catalogue no. 16140063, Thermo Fisher Scientific), 10% horse serum (catalogue no. 26050088, Thermo Fisher Scientific), 2.5 ng ml^−1^ basic fibroblast growth factor (catalogue no. PMG0035, Thermo Fisher Scientific), 1% penicillin-streptomycin (catalogue no. 15070063, Thermo Fisher Scientific) and 1% l-glutamine (catalogue no. 25030149, Thermo Fisher Scientific). Medium was refreshed the next day and cells were grown for 4 days until confluency. Cells were then differentiated for 2 days to myotubes in differentiation medium (DMEM, catalogue no. 11995065, Thermo Fisher Scientific) supplemented with 2% horse serum and 1% penicillin-streptomycin. Treatments were performed on day 4 of differentiation for 24 h. IM-PTEC cells isolated from immorto mice^70^ were a gift from A. Tammaro (Amsterdam UMC). Cells were maintained in DMEM/F12 medium (Gibco) with 10% FCS (Gibco), 5 μg ml^−1^ insulin and transferrin, 5 ng ml^−1^ sodium selenite (Gibco), 40 pg ml^−1^ triiodothyrionine (Sigma-Aldrich), 36 ng ml^−1^ hydrocortisone (Sigma-Aldrich) and 20 ng ml^−1^ epidermal growth factor (Sigma-Aldrich) with l-glutamine and antibiotics (both from Gibco) and mouse interferon-y (IFN-y) (10 ng ml^−1^; ProSpec) at 33 °C in 5% (v/v) CO_2_. Before being seeded for the experiments, cells were grown under restrictive conditions (37 °C 5% (v/v) CO_2_, in the absence of IFN-γ) for 7 days to downregulate SV40 large T antigen activity. Cell lines were regularly tested using a Rapid *Mycoplasma* Detection Kit (MycoGenie, Assay Genie).

Cells were treated with trigonelline iodide (catalogue no. MolPort-003-944-936, Molport) after internal quality control using mass spectrometry, showing more than 99.7% purity, NR chloride (ChromaDex), NA (catalogue no. N0761, Sigma-Aldrich), NAM (catalogue no. 72340, Sigma-Aldrich), NMN (catalogue no. N3501, Sigma-Aldrich), 2-OHNA (catalogue no. 251054, Sigma-Aldrich) and FK866 (catalogue no. F8557, Sigma-Aldrich) for the desired time. Unless otherwise stated, trigonelline treatment was performed at 1 mM. 2-OHNA and FK866 were used at 1 mM and 100 nM, respectively in all experiments, unless indicated otherwise. Hoechst H33342 solution (Invitrogen, Thermo Fisher Scientific) and JC-10 (catalogue no. ENZ-52305, Enzo Life Sciences) were used to assess mitochondrial function. Isotopically labelled trigonelline iodide ([^13^C,^2^H_3_]-trigonelline M + 4) was labelled with one ^13^C on the carbonyl group and three ^2^H on the nitrogen of the pyridine ring through custom synthesis and used in vitro and in vivo at the mentioned concentrations. Briefly, [carbonyl-^13^C]-NA (0.1 g, 0.8 mmol) was stirred in anhydrous ethanol (5 ml), followed by adding 200 μl (0.46 g, 3 mmol) of ^2^H_3_-methyl iodide at 23 °C. The mixture was stirred under reflux at 45–48 °C in an oil bath for 2 days. The oil bath was removed and the reaction mixture was allowed to cool down to room temperature. Ethanol was evaporated on a rotary evaporator. Yellow powder residue was first washed with ethanol (2× 1 ml) and then with diethyl ether (2 ml). The product was additionally dried under vacuum at 23 °C to give yellow solid (0.159 g, 74%; MS: 142.14 [M-I]+).

### NAD^+^ measurement

Cellular NAD^+^ levels were assessed using the BioVision NAD^+^/NADH Quantitation Colorimetric Kit (catalogue no. k337-100). Total NAD^+^ was normalized to the total number of cells. Quantification of NAD^+^ in tissue samples was performed using an enzymatic method adapted from Dall et al.^71^ with normalization to tissue weight. Analysis of the NAD^+^ metabolome in cells or in vivo samples was performed using high-resolution liquid chromatography–mass spectrometry as described below.

### Cellular assays

#### Cell death assays

Kinetic experiments of apoptosis were performed with the Incucyte ZOOM instrument (Essen BioScience). Cells were incubated with Incucyte Annexin-V Green (catalogue no. 4642), according to the supplier’s instructions. Four images per well were collected at the indicated time using a 10× objective and bandwidth filters (excitation: 440/80 nm; emission: 504/44 nm).

#### Mitochondrial function

For mitochondrial potential assessment, cells were seeded into a 96-well Sensoplate (catalogue no. 655090, Greiner) at a density of 12,000 cells per well in SKM-M. After 1 day, differentiation was induced by a medium change for 4 days; mitochondrial potential was performed at the desired time and with the desired treatment. Briefly, cells were incubated for 30 min in Krebs buffer with Hoechst staining and JC-10. Images were acquired using ImageXpress (Molecular Devices) using a 10× objective. The following filters were used: JC-10, FITC Filter Cube/TRITC Filter Cube; Hoechst, 4,6-diamidino-2-phenylindoleI Filter Cube. The total intensity of both FITC and TRITC fluorescence was recorded for each cell and was used to calculate a cellular fluorescence ratio: ratio per cell = log_2_ (∑ pixel intensity TRITC/∑ pixel intensity FITC). Once segmentation was completed, results were analysed with the KNIME software v.4.3.1.

The bioenergetic profiles of the cells were analysed using an XF96 extracellular flux analyzer (Seahorse Bioscience) as described. Briefly, cells were seeded in XF96 Cell Culture Microplates and the OCR was measured in Krebs buffer (NaCl 135 mM, KCl 3.6 mM, NaH_2_PO_4_ 0.5 mM, MgSO_4_ 0.5 mM, HEPES 10 mM, NaHCO_3_ 5 mM) supplemented with 10 mM glucose, 10 mM pyruvate and 2 mM glutamine. For substrate-driven OCR measurement in permeabilized HSMMs, the assay was performed in Seahorse XF Plasma Membrane Permeabilizer (Agilent Technologies) according to the manufacturer’s instructions. Briefly, cells were seeded in XF96 Cell Culture Microplates and the OCR was measured in mitochondrial assay solution (MAS buffer; mannitol 220 mM, sucrose 70 mM, KH_2_PO_4_ 10 mM, MgCl_2_ 5 mM, HEPES 2 mM, EGTA 1 mM, BSA 0.2% (w/v)) supplemented with ADP.

#### NAPRT knockdown in cells

To knockdown *NAPRT* in myotubes, 8,000 cells per well were seeded overnight in SKM-M medium. Cells were then infected with *NAPRT* shRNA or scrambled shRNA adenoviruses (SIRION Biotech) at 200 multiplicity of infection; cells were incubated for 48 h before initiating differentiation of myoblasts into myotubes. To check knockdown efficiency, cells were lysed in TriPure RNA reagent. Total RNA was transcribed to complementary DNA (cDNA) using the QuantiTect Reverse Transcription Kit (QIAGEN); mRNA expression was analysed using the LightCycler 480 instrument (Roche Diagnostics) and LightCycler 480 SYBR Green I Master Reagent (Roche Life Science). See Extended Data Table 6 for the list of primers used.

#### GPCR agonist assay

The GPR109A agonist assay was performed using the PathHunter β‐arrestin enzyme fragment complementation technology (Eurofins). Compounds were tested in duplicate in agonist mode and data were normalized to the maximal and minimal response observed in the presence of positive control and vehicle, respectively.

#### NAD^+^ precursor stability

The stability of the different precursors was assessed in human serum (catalogue no. H4522, Sigma-Aldrich) at 37 °C at the indicated time points. A liquid–liquid extraction was adopted from Giner et al.^72^ to assess the level of precursors or intermediates using the analytical methods described below.

### Animal studies

Studies and procedures in WT mice were approved by the Nestlé Ethical Committee (ASP-19-03-EXT), the Office Vétérinaire Cantonal Vaudois (VD2770 and VD3484) and the Animal Ethics Committee at The University of Melbourne (1914961.2). *Naprt* KO animal studies were approved by the Animal Experiment Committee at the University of Toyama (approval no. A2022MED-19) and were performed in accordance with the Guidelines for the Care and Use of Laboratory Animals at the University of Toyama, which are based on international policies.

In vivo treatments were performed with trigonelline monohydrate (Laurus Labs) after internal quality control using MS, showing more than 99.9% purity, or [^13^C,^2^H_3_]-trigonelline M + 4 iodide synthesized by the authors M.V.M. and M.E.M. for this study. Trigonelline was administered orally at a dose equimolar to 300 mg kg^−1^ of anhydrous trigonelline. For the in vivo tracer experiments, 12-week-old C57BL/6JRj male mice were given an acute dose of 300 mg kg^−1^ labelled [^13^C,^2^H_3_]-trigonelline M + 4 iodide by oral gavage; after 2 h and 24 h, blood, urine, muscle and liver were sampled. For chronic trigonelline supplementation, mice were fed with a standard chow diet alone or supplemented before pelleting with trigonelline monohydrate (Laurus Labs), at a dose delivering an average exposure of 300 mg kg^−1^ per day. Twenty-month-old aged C57BL/6JRj male mice were fed control or trigonelline diet (catalogue no. s8189, Ssniff) for 5 days, while young 12-week-old control and 20-month-old aged C57BL/6J male mice (The Jackson Laboratory) were fed control or trigonelline diet (AIN93M, Specialty Feeds) for 12 weeks. In the 12-week study, phenotypic characterization included body composition, using a whole-body composition analyser (LF50, Bruker), spontaneous physical activity of fine and ambulatory movements measured in Promethion metabolic cages (Sable Systems International), and grip strength assessed 1 week before the end of the study using a grip strength metre recording maximal force at peak tension before releasing the grasp. At the end of the study, tibialis anterior contractile muscle function was assessed under anaesthesia as described previously^73,74^. Briefly, maximum isometric tetanic force was determined from the plateau of a complete frequency–force relationship. Assessment of contraction-induced fatigue was determined by maximally stimulating muscles for an isometric contraction once every 4 s for 4 min and normalizing the decline of force to the baseline value. Force recovery was assessed after 5 min and 10 min rest after the initial stimulation period. A blood sample was subsequently obtained via cardiac puncture; after cardiac excision, skeletal muscles (tibialis anterior, extensor digitorum longus, soleus, plantaris, gastrocnemius, quadriceps, diaphragm strips) and organs (liver, kidneys) were surgically excised, weighed and snap-frozen for biochemical analysis or embedded and frozen in isopentane cooled in liquid nitrogen for immunohistochemical analysis. Plasma parameters were measured using the VetScan Equine Profile Plus.

For the *Naprt* KO study, animals were kept under a controlled temperature and humidity (25 °C, 50%) with standard light condition (a 12:12 h light–dark cycle) with free access to water and standard chow diet (CLEA Japan). *Naprt* KO mice of mixed sexes were obtained by crossing the heterogenic C57BL/6N *Naprt* KO mice described previously^40^ and treated with custom-synthesized trigonelline iodide at 8 weeks of age. Two hours later, tissues were collected and immediately frozen in liquid nitrogen and kept in −80 °C.

### Histological analyses

After cryosectioning, muscle sections were stained with haematoxylin & eosin for assessment of muscle architecture, CD31 to visualize capillaries, Van Gieson’s stain to identify collagen and fibrosis, SDH activity as a general marker of mitochondrial (oxidative) activity, and periodic acid–Schiff stain for glycogen content^75^. The muscle fibre cross-sectional area was measured after immunolabelling of laminin; fibre type was identified by staining myosin heavy chain I and myosin heavy chain IIa. Digital images of stained sections (four images per muscle section) were obtained using an upright microscope (20× objective) with a camera (Axio Imager D1, ZEISS) and AxioVision AC software (AxioVision AC, release 4.7.1, ZEISS) for acquisition. Myofibre cross-sectional area was quantified as described previously^76,77^.

### RNA and immunoblot analyses in rodent tissues

For qPCR of tissues from the *Naprt* KO study, RNA extraction was performed using the TRI Reagent (Sigma-Aldrich). The ReverTra Ace qPCR RT Master Mix with genomic DNA Remover (TOYOBO) was used to synthesize cDNA. Real-time PCR was performed using the THUNDERBIRD SYBR qPCR Mix (TOYOBO) on the Thermal Cycler Dice Real Time System II (Takara Bio). mRNA was quantified using the ∆∆^Ct^ method against *Rpl13a* as the reference gene. See Extended Data Table 6 for the list of primers used.

Oxidative phosphorylation complex expression was measured in isolated mitochondria by immunoblot using an antibody cocktail (1:250 dilution, catalogue no. 45-8099, Invitrogen) and anti-α-tubulin antibody (1:1,000 dilution, catalogue no. T6074, Sigma-Aldrich). Equal protein load (20 µg per well) and consistent gel transfer was verified by Revert Total Protein Stain (LI-COR). Immunoblots were imaged using a LI-COR IR imager and quantified with Image Studio Lite (v.5.0, LI-COR).

### Metabolomics analyses in cells and rodent tissues

Metabolomics analyses were conducted using a liquid–liquid extraction method adapted from Giner et al.^72^. For cell extraction, cells were scraped and extracted in a cold mixture of methanol:water:chloroform (5:3:5 (v/v)). Tissue extraction was performed with metal beads in pre-cooled racks (−80 °C) using a tissue mixer (TissueLyser II, QIAGEN). Whole blood and urine were directly extracted in cold methanol:water:chloroform (5:3:5 (v/v)). Isotopically labelled internal standards, including fully labelled ^13^C yeast extract and nicotinamide-d4, were included for data normalization. Additionally, isotopically labelled acyl-carnitines (NSK-B, Cambridge Isotope Laboratories) were added for normalization in cell metabolomics. After centrifugation, samples yielded an upper phase containing polar metabolites, a lower phase containing apolar metabolites, and a protein layer in between. The upper phase was dried and dissolved in 60% (v/v) acetonitrile:water for analysis. Protein layers from cells, liver and muscle samples were quantified using a bicinchoninic acid assay (Thermo Fisher Scientific) for normalization. HRLC–MS spectrometry was performed using hydrophilic interaction liquid chromatography (HILIC) analytical columns, such as HILICON iHILIC Fusion(P) or ZIC-pHILIC columns^77,78^. Separation was achieved using a linear solvent gradient with solvent A (H_2_O with 10 mM ammonium acetate and 0.04% (v/v) ammonium hydroxide, pH approximately 9.3) and solvent B (acetonitrile). The eluting NAD^+^ metabolites were analysed using an Orbitrap Fusion Lumos mass spectrometer (Thermo Fisher Scientific) with a heated electrospray ionization source. Data processing and instrument control were performed using the Xcalibur v.4.1.31.9 software (Thermo Fisher Scientific). Quantitative trigonelline measurement in tissues was performed using the same liquid–liquid extraction method, while body fluids (plasma and urine) were extracted using ACN:MeOH:H_2_O (−20 °C) as described by Li et al.^79^. A labelled amino acid mix (NSK-A1, Cambridge Isotope Laboratories) served as an internal standard. Leucine-d4 was used as an internal standard for normalization. Trigonelline concentrations were determined using calibration curves ranging from 10 µM to 1,000 µM. For the tracer experiments, the same methods were used and the relative abundance and enrichment of isotopologues of NAD metabolites were calculated by dividing the area of each isotopologue by the sum area of all isotopologues. In the *Naprt* KO rodent study, metabolite extraction and NAD^+^ metabolomics were performed as described previously^80^. Tissues were grounded in a 50% methanol 50% water mixture using a multi-bead shocker (Yasui Kikai). Metabolites were analysed using an Agilent 6460 Triple Quad Mass Spectrometer coupled with an Agilent 1290 HPLC system. Trigonelline and other NAD^+^ metabolites were detected using specific transitions; data analysis was conducted using the MassHunter Workstation Quantitative Analysis software (Agilent Technologies).

### *C. elegans* experiments

WT hermaphrodite Bristol worms (N2) and GFP-tagged myosin worms^81^ were treated with trigonelline chloride (catalogue no. T5509, Sigma-Aldrich) and NR chloride from day 1 of adulthood. Nematodes were cultured at 20 °C on nematode growth medium (NGM) agar plates seeded with *Escherichia coli* strain OP50. Trigonelline and the other precursors were added to the NGM medium at a final concentration of 1 mM just before pouring the plates, unless otherwise stated. An enzymatic method adapted from Dall et al.^71^ was used to measure NAD^+^ content in worms. Fifty to 100 worms were collected per biological replicate and NAD^+^ levels were normalized on the protein content. The bacterial feeding RNAi experiments were carried out as described^82^. RNAi was performed using the clones *sir-2.1* (R11A8.4) and *nprt-1* (Y54G2A.17) from Geneservice.

#### Lifespan

Worm lifespan tests were performed using 90–100 animals per condition and scored manually every other day, as described previously^83^. Treatments and experimental measurements were started on day 1 of WT N2 worm adulthood, in a regimen of chronic exposure during the entire study. Statistical significance was calculated using the log-rank (Mantel–Cox) method.

#### Mobility assay

*C. elegans* spontaneous mobility was measured using the Movement Tracker software (v.1)^84^. For paralysis scoring, 45–60 worms per condition were manually scored for mobility after poking. Worms that did not respond to any repeated stimulation were scored as dead. Results are representative of data obtained from at least two independent experiments.

#### RNA analyses

A total of approximately 6,000 worms per condition, divided into six biological replicates, was recovered in M9 buffer from the NGM plates at day 2 of adulthood and lysed in the TriPure RNA reagent. Total RNA was transcribed to cDNA using the QuantiTect Reverse Transcription Kit. Expression of selected genes was analysed using the LightCycler 480 system and LightCycler 480 SYBR Green I Master Reagent. For worms, two housekeeping genes were used to normalize the expression data, that is, actin (*act-1*) and peroxisomal membrane protein 3 (*pmp-3*). See Extended Data Table 6 for the list of primers used.

#### Muscle integrity imaging

Imaging of muscle structure was performed on RW1596 *myo-3* (*st386)* worms^55^. Trigonelline treatment started on day 1 of adulthood, in a regimen of chronic exposure until day 11. Worms were immobilized with a 7.5-mM solution of tetramisole hydrochloride (Sigma-Aldrich) in M9 and mounted on 2% agarose pads on glass slides. Confocal images were acquired with Leica SP8 inverse STED 3X (Leica Microsystems) under non-saturating exposure. Myofibre integrity scoring was quantified as described by Dhondt et al.^85^ on a total of six worms per group and 9–31 muscle cells per group.

#### Oxygen consumption assays

Oxygen consumption was measured in N2 worms treated from embryo to L4 with trigonelline using the Seahorse XF96 system (Seahorse Bioscience) as described previously^86^. Respiration rates were normalized to the number of worms in each well and averaged over five measurements.

#### Mitochondrial DNA quantification

Worms were lysed in 2 μl lysis buffer (50 mM KCl, 10 mM Tris pH 8.3, 2.5 mM MgCl_2_, 0.45% NP-40, 0.45% Tween 20, 0.01 gelatin, 100 μg ml^−1^ proteinase K (added before use)) and heated at 65 °C for 60 min followed by inactivation at 90 °C for 15 min. Worm lysates were diluted 50× with diethyl pyrocarbonate water for the SYBR Green qPCR reaction using a LightCycler 480 SYBR Green I Master Kit assay. Relative values for *nd-1* and *act-3* (Extended Data Table 6) were compared in each sample to generate a ratio representing the relative level of mitochondrial DNA per nuclear genome. Experiments were performed on at least ten independent biological samples.

### Statistics

All statistical analyses were performed using Prism v.9 (GraphPad Software) or R as described in the figure legends. The statistical methods for the transcriptomic analyses of muscle biopsies have been reported previously^3^. Data distributions plotted as box plots represent the 25th percentile, the median and 75th percentile, with the whiskers extending from the quartiles to the smallest or biggest value within 1.5 times the interquartile range. Associations between two continuous variables were determined using Spearman rank correlations. For statistical comparisons of two conditions, a two-tailed, unpaired Student’s *t*-test was used. For comparisons of more than two groups, data were analysed with a one-way ANOVA followed by Šídák’s multiple comparisons post hoc test or a two-way ANOVA, unless specified otherwise in the figure legends. The normality of each readout was assessed based on historical values of the laboratory for this readout. All data represent the mean ± s.e.m. NS indicates results that were not statistically significant; *P < 0.05, **P < 0.01, ***P < 0.001 and ****P < 0.0001 were considered statistically significant.

### Reporting summary

Further information on research design is available in the Nature Portfolio Reporting Summary linked to this article.

### Supplementary information

Reporting Summary

### Source data

Source Data Fig. 1 Individual values and statistics.

Source Data Fig. 2 Individual values and statistics.

Source Data Fig. 3 Individual values and statistics.

Source Data Fig. 4 Individual values and statistics.

Source Data Extended Data Fig. 1 Individual values and statistics.

Source Data Extended Data Fig. 2 Individual values and statistics.

Source Data Extended Data Fig. 3 Individual values and statistics.

Source Data Extended Data Fig. 4 Individual values and statistics.

Source Data Extended Data Fig. 4 Unprocessed immunoblots.

## Figures and Tables

**Extended Data Table 1:**
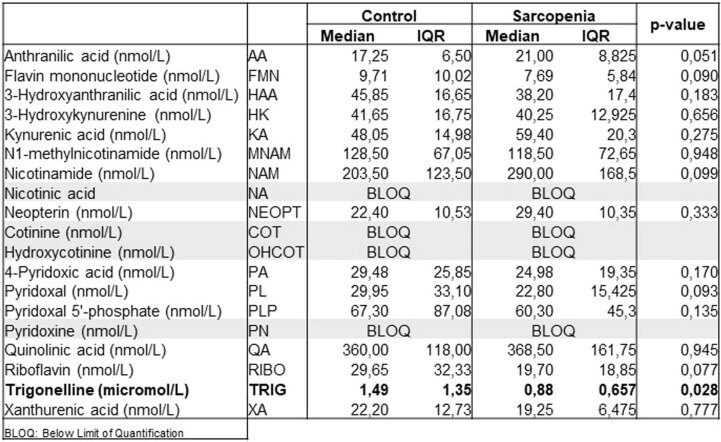
Profiling of serum metabolites of kynurenine/NAD^+^ metabolism in the Singapore Sarcopenia Study of MEMOSA

The serum metabolites of kynurenine/NAD^+^ were measured in the Singapore sarcopenia study of MEMOSA using LC–MS/MS (*n* = 40).

**Extended Data Table 2:**
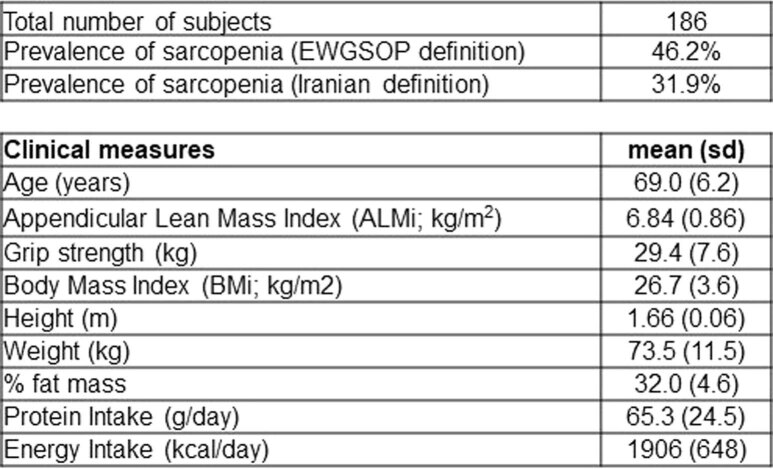
Clinical characterization of the Bushehr Elderly Health Cohort

Clinical parameters were measured in 186 older men of the Bushehr nutritional epidemiology study aged 60 years and older. EWGSOP: European Working Group on Sarcopenia in Older People.

**Extended Data Table 3:**
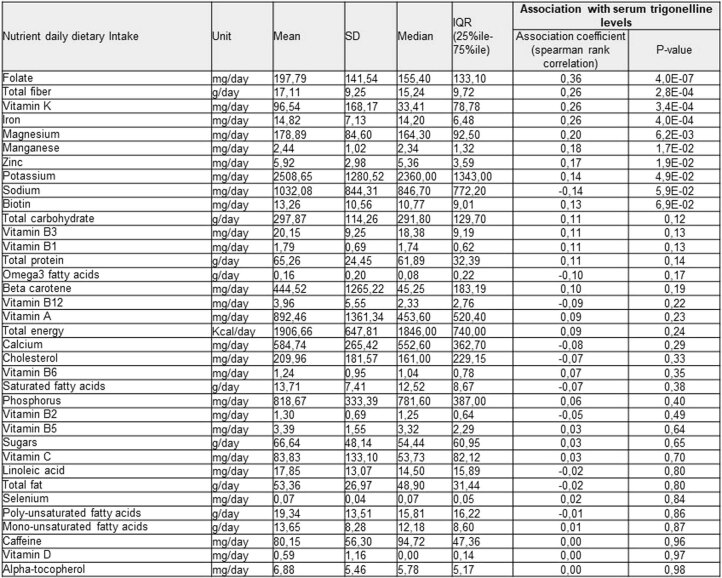
Association of dietary intake with serum trigonelline levels in the Bushehr Elderly Health Cohort

Dietary intake assessed using 24-h recall food questionnaire in the Bushehr Elderly Health Cohort was associated with serum trigonelline levels measured with LC–MS/MS and analysed with Spearman rank correlation.

**Extended Data Table 4:**
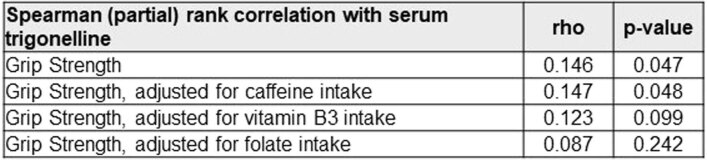
Association of grip strength with serum trigonelline levels corrected for dietary intake in the Bushehr Elderly Health Cohort

Handgrip strength in the Bushehr Elderly Health Cohort was associated with serum trigonelline levels measured with LC–MS/MS and corrected for dietary intake assessed with a 24-h recall food questionnaire and analysed with a Spearman partial rank correlation.

**Extended Data Table 5:**
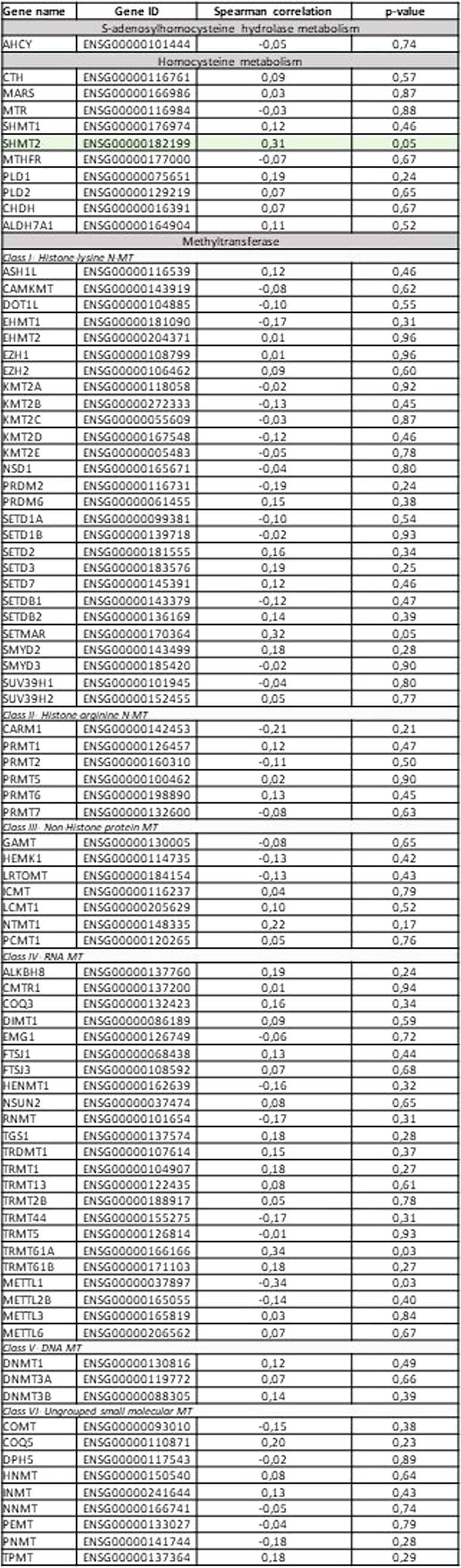
Association of serum trigonelline levels with muscle mRNA expression of demethylase and methyltransferase genes in the Singapore Sarcopenia Study

Serum trigonelline levels measured with LC–MS/MS in the Singapore Sarcopenia study of MEMOSA was associated with gene expression measured using RNA sequencing in muscle biopsies, analysed with a Spearman rank correlation, filtered for genes expressed in skeletal muscle and annotated with methyltransferase or demethylase activity.

**Extended Data Table 6:**
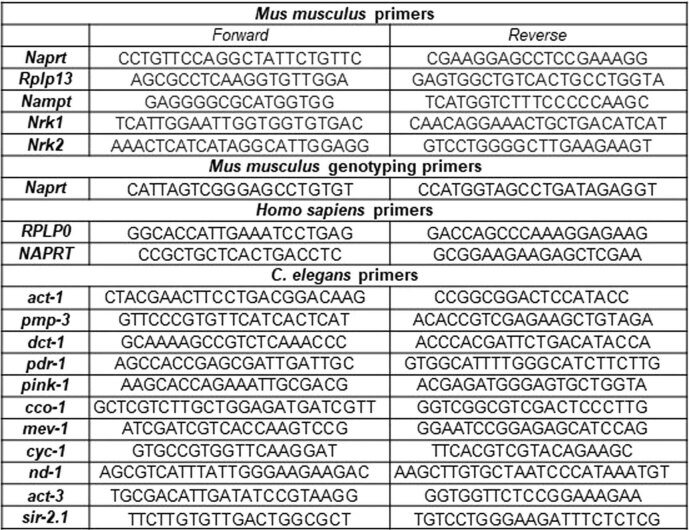
qPCR primers

List of qPCR primers used to detect *C. elegans*, mice and human gene expression in this study (5′ > 3′).

## Data Availability

The unprocessed transcriptomic data of this study have been deposited in the Gene Expression Omnibus under accession no. GSE111016. Clinical data cannot be made openly available because of study ethical approvals. These data can be provided on justified request subject to appropriate approvals, after a formal application to the Oversight Group of the different cohorts through their respective corresponding author. Source data are provided with this paper.
