# Supplementary material for: Trigonelline is an NAD+ precursor that improves muscle function during ageing and is reduced in human sarcopenia
Source: Nat Metab. 2024 Mar 19;6(3):433–47. doi: 10.1038/s42255-024-00997-x (PMC10963276; doi:10.1038/s42255-024-00997-x)
Supplement: Supplementary file 1 — Reporting Summary [file 42255_2024_997_MOESM1_ESM.pdf]

nature portfolio

Corresponding author(s): Vincenzo Sorrentino
Jerome Feige

Last updated by author(s): Dec 26, 2023

Reporting Summary

Nature Portfolio wishes to improve the reproducibility of the work that we publish. This form provides structure for consistency and transparency in reporting. For further information on Nature Portfolio policies, see our Editorial Policies and the Editorial Policy Checklist.

Statistics

For all statistical analyses, confirm that the following items are present in the figure legend, table legend, main text, or Methods section.

n/a	Confirmed	
☐	☒	The exact sample size (*n*) for each experimental group/condition, given as a discrete number and unit of measurement
☐	☒	A statement on whether measurements were taken from distinct samples or whether the same sample was measured repeatedly
☐	☒	The statistical test(s) used AND whether they are one- or two-sided<br>*Only common tests should be described solely by name; describe more complex techniques in the Methods section.*
☐	☒	A description of all covariates tested
☐	☒	A description of any assumptions or corrections, such as tests of normality and adjustment for multiple comparisons
☐	☒	A full description of the statistical parameters including central tendency (e.g. means) or other basic estimates (e.g. regression coefficient) AND variation (e.g. standard deviation) or associated estimates of uncertainty (e.g. confidence intervals)
☐	☒	For null hypothesis testing, the test statistic (e.g. *F*, *t*, *r*) with confidence intervals, effect sizes, degrees of freedom and *P* value noted<br>*Give P values as exact values whenever suitable.*
☒	☐	For Bayesian analysis, information on the choice of priors and Markov chain Monte Carlo settings
☒	☐	For hierarchical and complex designs, identification of the appropriate level for tests and full reporting of outcomes
☐	☒	Estimates of effect sizes (e.g. Cohen's *d*, Pearson's *r*), indicating how they were calculated

Our web collection on statistics for biologists contains articles on many of the points above.

Software and code

Policy information about availability of computer code

Data collection	RNA seq data were collected using the vendor's software Illumina HiSeq 2500
Data analysis	All statistical analyses concerning RNA-seq data were conducted in R version 3.3.3 using relevant Bioconductor packages (e.g. limma 3.30.13, edgeR 3.16.5). Pathway enrichment analysis was performed using CAMERA querying gene sets annotated in MSigDB v5.2. P<br>For all the other experimental data, statistical analysis was carried out using GraphPad Prism 9 or R. The statistical methods used for each analysis are mentioned in the figure legends.<br>For metabolomics: the software Xcalibur v4.1.31.9 (Thermo Scientific) or the MassHunter Workstation-Quantitative Analysis (Version B.05.00, Agilent technologies, Santa Clara, CA, USA) were used for instrument control and data processing of isotopically labelled and unlabelled metabolites.<br>For qPCR analysis, the LightCycler480 system and software (Roche) were used.<br>For mitochondrial membrane potential images analysis: Images were acquired using the ImageXpress (Molecular Device) using 10x objective. The following filters were used: JC-10, FITC Filter Cube/ TRITC Filter Cube; Hoechst, DAPI Filter Cube. One site per well was acquired with a 10x objective, and images were segmented to recognize cells using "Mitochondrial Potential-Regular Segmentation" analysis. The total intensity of both FITC and TRITC fluorescence is recorded for each cell and is used to calculate a cellular fluorescent ratio: Ratio per cell = log2 (∑pixel intensityTRITC/∑pixel intensityFITC). Once segmentation was completed, results were analyzed using KNIME software version 4.3.1.<br>For histology images: digital images of stained sections were obtained using an upright microscope (20X objective) with camera (Axio Imager D1, Carl Zeiss, Wrek Göttingen, Germany), controlled by AxioVision AC software (AxioVision AC Rel. 4.7.1, Carl Zeiss Imaging Solutions, Wrek, Göttingen, Germany). Images were quantified using AxioVision 4.7.1 software for average fiber CSA.<br>The bioenergetic profiles of cells were analyzed by an XF96 extracellular flux analyzer (Seahorse Biosciences, North Billerica, MA, USA).<br>Mobility of worms: C. elegans movement analysis was performed using the Movement Tracker software (Mouchiroud, L. et al. doi:10.1002/cpns.17 (2016), version 1.

Myofibers integrity scoring in C. elegans was performed with the method described by Dhondt et al. (Dhondt, I. et al. Dis Model Mech 14, doi:10.1242/dmm.049169 (2021))

For manuscripts utilizing custom algorithms or software that are central to the research but not yet described in published literature, software must be made available to editors and reviewers. We strongly encourage code deposition in a community repository (e.g. GitHub). See the Nature Portfolio guidelines for submitting code & software for further information.

Data

Policy information about availability of data

All manuscripts must include a data availability statement. This statement should provide the following information, where applicable:
- Accession codes, unique identifiers, or web links for publicly available datasets
- A description of any restrictions on data availability
- For clinical datasets or third party data, please ensure that the statement adheres to our policy

All original data, uncropped gels and all the individual p values presented herein are included in the Source data files. The unprocessed transcriptomic data of this study have been deposited in the Gene Expression Omnibus under accession number GSE111016 . Clinical data cannot be made openly available from study ethical approvals. These data can be provided upon justified request subject to appropriate approvals, after a formal application to the Oversight Group of the different cohorts through their respective corresponding author.

Human research participants

Policy information about studies involving human research participants and Sex and Gender in Research.

Reporting on sex and gender	Only men were included in the discovery clinical studies. This was decided because of the exploratory nature of the study, small sample size and past experience of lower enrollment of older women in studies where muscle biopsies are collected. For the replication study, gender was matched to the discovery study for powering purposes as the blood levels of many micronutrients and their association with body composition are sex-specific (Konz et al, Front Physiol 219).
Population characteristics	Singapore Sarcopenia Study (SSS). Forty Chinese descent male aged 65-79 years with or without sarcopenia were recruited. Self-reported ethnicity was collected during the inclusion visit and weight and height were measured to the nearest 0.1kg and 1cm, respectively. Clinical charcteristics of the cohort have bene reported in Migliavacca et al, Nature Comms 2019.<br>Bushehr nutritional epidemiology study: 186 older men aged 60 years and above randomly selected from the population-based BEH prospective cohort study conducted in Bushehr, a southern province of Iran. The general clinical characteristics (age, weight, height, BMI...) of the Bushehr Elderly Health Cohort are reported in table S2.
Recruitment	Singapore Sarcopenia Study (SSS). Forty male subjects with and without a diagnosis of sarcopenia of comparable age group were recruited prospectively for the MEMOSA project following screening with defined inclusion/exclusion criteria. Screening was performed via two existing studies on healthy community-dwelling older men in Singapore (Singapore Sarcopenia Group and Aging in a Community Environment Study).<br>Bushehr nutritional epidemiology study in older people: 186 older men aged 60 years and above participating in the second stage of the Bushehr elderly health (BEH) program were randomly selected from the population-based prospective cohort study conducted in Bushehr, a southern province of Iran.
Ethics oversight	Participants were recruited from two studies on healthy community-dwelling older men in Singapore (Singapore Sarcopenia Group and Aging in a Community Environment Study [ACES]). The National Healthcare Group Domain-Specific Research Board (NHG DSRB) approved the study, reference number 2014/01304, and each participant gave written informed consent.<br>The Bushehr nutritional epidemiology study was approved by the Research Ethics Committee of the Endocrinology & Metabolism Research, Tehran University of Medical Sciences, under reference TUMS.EMRI.REC.1394.0036, and each participant gave written informed consent.<br>Local sample analyses were approved by the cantonal ethics commission for human research (CER-VD) in Vaud, Switzerland under reference 490/14.

Note that full information on the approval of the study protocol must also be provided in the manuscript.

Field-specific reporting

Please select the one below that is the best fit for your research. If you are not sure, read the appropriate sections before making your selection.

☒ Life sciences ☐ Behavioural & social sciences ☐ Ecological, evolutionary & environmental sciences

For a reference copy of the document with all sections, see nature.com/documents/nr-reporting-summary-flat.pdf

Life sciences study design

All studies must disclose on these points even when the disclosure is negative.

Sample size	For the human studies, 40 serum samples were analyzed in the MEMOSA Singapore cohort (SSS) based on available samples from the previously approved study design (Migliavacca et al. Nature Communication 2019, doi:10.1038/s41467-019-13694-1 (2019)). For the Bushehr

study, serum samples from 186 older men were analyzed.

For the preclinical and in vitro studies, sample size was estimated based on (I) the variability observed in pilot studies or in previous related experiments, (II) the expected effect size, and (III) the probability to detect a minimal significance level of 0.05.For all animal experiments, the lowest number of animals that were expected to be required to obtain statistical significance was chosen. For aged mice more animals were included due to the expected higher (natural) mortality.

Data exclusions

For the human studies, only remaining samples from the previously approved MEMOSA study with sufficient muscle biopsy material were analyzed in the SSS cohort for NAD+ levels (Migliavacca et al. Nature Communication 2019, doi:10.1038/s41467-019-13694-1 (2019)).

For the preclinical studies, mice that showed weight loss >20%, were found dead in cages or displayed signs of wound infection and inflammation, were excluded from the study.

Replication

The RNAseq results from SSS human muscle biopsies have been previously validated using Nanostring on the 50 most regulated genes in sarcopenia (See Migliavacca et al, Nature Comms 2019))
The results obtained in cellular and worm experiments were replicated in 2-4 independent experiments. Mouse results were obtained from 3 independent experiments, including different locations.

Randomization

For the human studies, given our experimental setting, groups were not randomized.
Animals were randomized by body weight within experimental groups. All animals of the same gender and age were considered similar

Blinding

For the human studies, people conducting experiments in the laboratories were blind to the sample labels.
For the chronic mouse study, researchers conducting the experiments were blinded to the treatment during the phenotyping tests, including metabolic cage, grip strength, TA contractile function and histology. For worm experiments, researchers performing the assays were blinded to the treatments. For all the other animal and cellular studies, blinding was not formally performed as measures reported are quantitative.

Reporting for specific materials, systems and methods

We require information from authors about some types of materials, experimental systems and methods used in many studies. Here, indicate whether each material, system or method listed is relevant to your study. If you are not sure if a list item applies to your research, read the appropriate section before selecting a response.

Materials & experimental systems

n/a	Involved in the study	
☐	☒	Antibodies
☐	☒	Eukaryotic cell lines
☒	☐	Palaeontology and archaeology
☐	☒	Animals and other organisms
☐	☒	Clinical data
☒	☐	Dual use research of concern

Methods

n/a	Involved in the study	
☒	☐	ChIP-seq
☒	☐	Flow cytometry
☒	☐	MRI-based neuroimaging

Antibodies

Antibodies used

Mouse monoclonal OXPHOS antibody cocktail (Abcam 110412, 1/250); Mouse monoclonal Tubulin antibody (Sigma T6074, 1/1000); CD45 (Invitrogen, #MCD4528, 1/25), CD31 (Invitrogen, #RM5228, 1/25), CD11b (Invitrogen, #RM2828, 1/25), CD34 (BD Biosciences, #560238, 1/60), Ly-6A/E (BD Biosciences, #561021, 1/150) and α7-integrin (R&D Systems, #FAB3518N, 1/30).

Validation

- Mouse monoclonal OXPHOS antibody cocktail has been validated by the manufacturer in two dimension Blue Native PAGE analysis of fibroblasts that are normal and complex I deficient. Antibody profiles are available on Abcam website and it has been used for western blot applications in human samples for the following publications:
Newman LE et al. The ARL2 GTPase is required for mitochondrial morphology, motility, and maintenance of ATP levels. PLoS One 9:e99270 (2014).
Sánchez E et al. LYRM7/MZM1L is a UQCRFS1 chaperone involved in the last steps of mitochondrial Complex III assembly in human cells. Biochim Biophys Acta (2012).
- Mouse monoclonal Tubulin antibody (T6074 Sigma), validated also in the following publications on the Sigma website:
Increased expression of αTubulin is associated with poor prognosis in patients with pancreatic cancer after surgical resection.
Chao L, et al.Oncotarget, 7(37), 60657-60664 (2016)
LeDizet M and Piperno G J Cell Biol., 104, 13-22 (1986)
Regulation of alternative splicing by the core spliceosomal machinery.
Arneet L Saltzman et al.Genes & development, 25(4), 373-384 (2011-02-18)
- CD45 antibody was verified by Knockout to ensure that the antibody binds to the antigen stated. Antibody profiles are available on Thermoscientific website with applications in:
Vora P et al. The Rational Development of CD133-Targeting Immunotherapies for Glioblastoma. Cell stem cell (2020);
Cremer A et al. Resistance Mechanisms to SYK Inhibition in Acute Myeloid Leukemia. Cancer discovery (2020).
-CD31 antibody was verified by staining C57BL/6 mouse spleen cells and applying flow citometry. Antibody profiles are available on Thermoscientific website with applications in:
Parker E et al. indlimb Immobilization Increases IL-1 and Cdkn2a Expression in Skeletal Muscle Fibro-Adipogenic Progenitor Cells: A Link Between Senescence and Muscle Disuse Atrophy. Frontiers in cell and developmental biology (2022);

Madaro L et al. Macrophages fine tune satellite cell fate in dystrophic skeletal muscle of mdx mice. PLoS genetics (2019)
-CD11b was verified similarly as above for flow citometry, and applied in several studies:
Vogel A et al. JAK1 signaling in dendritic cells promotes peripheral tolerance in autoimmunity through PD-L1-mediated regulatory T cell induction. Cell reports (2022);
Deng C et al. BECN2 (beclin 2) Negatively Regulates Inflammasome Sensors Through ATG9A-Dependent but ATG16L1- and LC3-Independent Non-Canonical Autophagy. Autophagy (2022).
-CD34 is a FITC-conjugated monoclonal rat antibody, supplied by BD Biosciences, raised against Hematopoietic progenitor cell antigen CD34 (Mouse), cited in 66 publications:
Zhang, Z et al. Exosomes derived from human umbilical cord mesenchymal stem cells alleviate Parkinson's disease and neuronal damage through inhibition of microglia. Neural Regeneration Research (2023);
Lv, Y. Distinct response of adipocyte progenitors to glucocorticoids determines visceral obesity via the TEAD1-miR-27b-PRDM16 axis. Obesity (Silver Spring, Md.) (2023).
-Ly-6A/E is a PE-Cy7-conjugated monoclonal rat antibody, supplied by BD Biosciences, raised against Lymphocyte antigen 6A-2/6E-1 (Mouse), cited in 15 publications:
Vanneste, D., Bai, Q., et al. MafB-restricted local monocyte proliferation precedes lung interstitial macrophage differentiation. Nature Immunology (2023);
Eislmayr, K., Bestehorn, A., et al. Nonredundancy of IL-1α and IL-1β is defined by distinct regulation of tissues orchestrating resistance versus tolerance to infection. Science Advances (2022).
- α7-integrin is an Alexa Fluor 700-conjugated monoclonal rat antibody, supplied by R&D Systems, used in:
Parker, E., Khayrullin, A., et al. Hindlimb Immobilization Increases IL-1β and Cdkn2a Expression in Skeletal Muscle Fibro-Adipogenic Progenitor Cells: A Link Between Senescence and Muscle Disuse Atrophy. In Frontiers in Cell and Developmental Biology (2022);
Der Vartanian, A., Quétin, M., et al. PAX3 Confers Functional Heterogeneity in Skeletal Muscle Stem Cell Responses to Environmental Stress. Cell Stem Cell (2019).

Eukaryotic cell lines

Policy information about cell lines and Sex and Gender in Research

Cell line source(s)

- Human primary myoblasts (HSMM) (Lonza, CC-2580).
- Primary myotubes from healthy vs sarcopenic subjects were derived from muscle biopsies from participants in the Hertfordshire Sarcopenia Study Extension (HSSe) with approval from the Hertfordshire Research Ethics Committee (doi:10.1002/jcsm.12876 (2022)).
- Primary myotubes from aged male C57BL/6J mice (24 months) were generated from freshly sorted muscle stem cells isolated by flow cytometry.
- HepG2 (HB-8065) and C2C12 (CRL-1772) cell lines were from ATCC, Manassas, VA, USA.
- IM-PTEC cells isolated from Immorto mice (doi:10.1681/asn.2010040423 (2011)) were a gift from Dr. Alessandra Tammaro (Amsterdam UMC, the Netherlands).

Authentication

-All cell lines were autheticated by direct purchasing from ATCC.
- Human skeletal muscle cells were tested for myogenicity via myogenic and fibroblast markers, and further quality checked for their ability to differentiate into mature myotubes.
- Primary mouse myoblasts from hindlimb muscles from were rapidly collected, minced, and digested with 2.5 U/ml Dispase II (Sigma), 0.2% Collagenase B (Sigma) and 5 mM MgCl2 at 37°C. The preparation was then filtered sequentially through 100 micron and 30 micron filters and cells were incubated at 4°C for 30 min with antibodies against CD45 (Invitrogen, #MCD4528, 1/25), CD31 (Invitrogen, #RM5228, 1/25), CD11b (Invitrogen, #RM2828, 1/25), CD34 (BD Biosciences, #560238, 1/60), Ly-6A/E (BD Biosciences, #561021, 1/150) and α7-integrin (R&D Systems, #FAB3518N, 1/30). MuSCs identified as CD31-/CD11b-/CD45-/Sca1-/CD34+/Integrin α7+ were isolated with a Beckman Coulter Astrios Cell sorter.

Mycoplasma contamination

Cells were regularly tested for mycoplasma using a Rapid Mycoplasma Detection Kit (MycoGenie from AssayGenie).

Commonly misidentified lines
(See ICLAC register)

No commonly misidentified cell lines were used in the study.

Animals and other research organisms

Policy information about studies involving animals; ARRIVE guidelines recommended for reporting animal research, and Sex and Gender in Research

Laboratory animals

- 12-week and 20-month-old male C57BL/6J (Janvier, C57BL/6JRj) and C57BL/6J Jackson Laboratory Maine, USA) mice were included in this study
- 8 weeks-old Naprt KO mice of mixed genders were obtained by crossing heterogenic C57BL/6N Naprt KO mice described previously(doi:10.1038/s41467-021-27080-3 (2021)).
- For C. elegans studies the strains used in this study were the wild-type Bristol N2 and the myo-3::GFP strain RW1596. The worm studies were completed at several ages: Day 1, Day 8 or Day 11 of adulthood, or for lifespans experiments animals were followed till age of death (as indicated in the figure legends and Data Source files)

Wild animals

No wild animals were used in the study.

Reporting on sex

C57BL6 WT mice used in the study were males since the commercial supplier of aged mice could only provide males. NAPRT WT and KO mouse studies included both male and females.

Field-collected samples	No field collected samples were used in the study.
Ethics oversight	All procedures were approved by the Nestlé Ethical committee (ASP-19-03-EXT), the Office vétérinaire cantonal Vaudois (VD2770 and VD3484), and the Animal Ethics Committee at The University of Melbourne (1914961.2).<br>Naprt KO animal studies were approved by the Animal Experiment Committee at the University of Toyama (approval A2022MED-19), and were performed in accordance with the Guidelines for the Care and Use of Laboratory Animals at the University of Toyama, which are based on international policies.

Note that full information on the approval of the study protocol must also be provided in the manuscript.

Clinical data

Policy information about clinical studies

All manuscripts should comply with the ICMJE guidelines for publication of clinical research and a completed CONSORT checklist must be included with all submissions.

Clinical trial registration	The Memosa study was approved by the Singapore National Healthcare Group Domain-Specific Research Board (NHG DSRB) under reference number.<br>The Bushehr study was approved by the Research Ethics Committee of the Endocrinology & Metabolism Research, Tehran University of Medical Sciences, under reference TUMS.EMRI.REC.1394.0036. Local sample analyses were approved by the cantonal ethics commission for human research (CER-VD) in Vaud, Switzerland under reference 490/14.
Study protocol	2014/01304
Data collection	Described in Migliavacca et al, Nature Comms 2019
Outcomes	Described in Migliavacca et al, Nature Comms 2019
