# Supplementary figures and images for: Trigonelline is an NAD+ precursor that improves muscle function during ageing and is reduced in human sarcopenia
Source: Nat Metab. 2024 Mar 19;6(3):433–47. doi: 10.1038/s42255-024-00997-x (PMC10963276; doi:10.1038/s42255-024-00997-x)

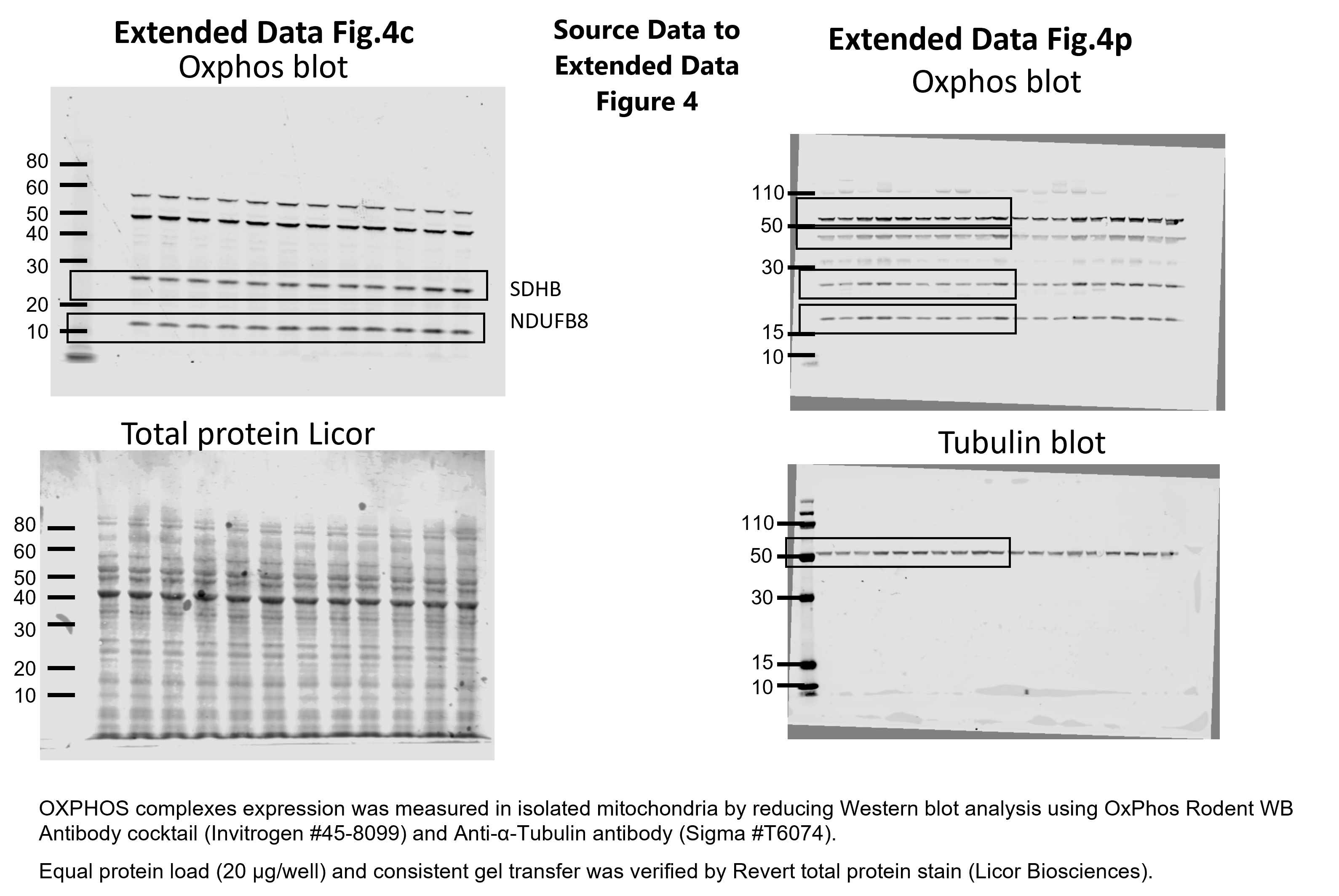

Supplement: Supplementary file 10 — Unprocessed immunoblots. [file 42255_2024_997_MOESM10_ESM.tif]
